# Supplementary material for: Modeling microbial metabolic trade-offs in a chemostat
Source: PLoS Comput Biol. 2020 Aug 28;16(8):e1008156. doi: 10.1371/journal.pcbi.1008156 (PMC7482850; doi:10.1371/journal.pcbi.1008156)
Supplement: S1 Appendix — (DOCX) [file pcbi.1008156.s001.docx]

**Modeling microbial metabolic trade-offs in a chemostat**

**S1 Appendix**

Zhiyuan Li^1,2,3,4^, Bo Liu^4,5^, Sophia Hsin-Jung Li^6^, Christopher G. King^7^, Zemer Gitai^6^, and Ned S. Wingreen*^6,8^

^1.^ Center for Quantitative Biology, Peking University, Beijing, China.

^2.^ Peking-Tsinghua Center for Life Sciences, Peking University, Beijing, China.

^3.^ Center for the Physics of Biological Function, Princeton University, Princeton, New Jersey, USA.

^4.^ Princeton Center for Theoretical Science, Princeton University, Princeton, New Jersey, USA.

^5.^ Yuanpei College, Peking University, Beijing, China.

^6.^ Department of Molecular Biology, Princeton University, Princeton, New Jersey, USA.

^7.^ Department of Physics, Princeton University, Princeton, New Jersey, USA.

^8.^ Lewis-Sigler Institute for Integrative Genomics, Princeton University, Princeton, New Jersey, USA.

* Email: wingreen@princeton.edu

Table of Contents

[Total RNA and total protein measurements 3](#_Toc42902629)

[Supplemental Table 1: Symbols 3](#_Toc42902630)

[Metabolic model and resource allocation strategy 4](#_Toc42902631)

[Dynamic equations for a single species in a chemostat 4](#_Toc42902632)

[Species-specific steady state 4](#_Toc42902633)

[*Growth contour (GC)* 5](#_Toc42902634)

[*Flux-balance curve (FB)* 5](#_Toc42902635)

[*Supply line (SL)* 5](#_Toc42902636)

[Dynamic equations for multiple species in a chemostat 6](#_Toc42902637)

[Multiple species steady state 6](#_Toc42902638)

[Invasion 7](#_Toc42902639)

[*Invasion zone* 7](#_Toc42902640)

[Fitness landscape 7](#_Toc42902641)

[Non-invasible/optimal/evolutionarily stable strategies 8](#_Toc42902642)

[*1. Maximal growth rates and maximizing strategies* 8](#_Toc42902643)

[*2. Maximal growth contour* 8](#_Toc42902644)

[*3. Non-invasible strategy* 8](#_Toc42902645)

[*4. Evolutionarily stable coexistence at the discontinuous points of the maximal growth contour* 9](#_Toc42902646)

[Metabolic models 9](#_Toc42902647)

[*1. Metabolic model with substitutable nutrients* 9](#_Toc42902648)

[*2. Metabolic model with two essential nutrients* 12](#_Toc42902649)

[*3. Metabolic model with substitutable nutrients that require assimilation* 14](#_Toc42902650)

[*4. Metabolic model with essential nutrients that can be interconverted* 15](#_Toc42902651)

[*5. Metabolic model with multiple energy generating steps* 17](#_Toc42902652)

[Dynamic equations for multiple species in a chain of chemostats 18](#_Toc42902653)

## **Total RNA and total protein measurements**

The methods for total RNA and protein measurements shown in Fig S2 are described in [1].

## **Supplemental Table 1: Symbols**

| Chemostat parameters | |
| --- | --- |
| $\vec{c}_{\mathrm{supply}}=\left( c_{1,\text{ supply}},c_{2,\text{ supply}},\ldots c_{p,\text{ supply}} \right)$ | Nutrient supply. $c_{i,\text{ supply}}$ is the concentration of the $i$-th nutrient in the supply. |
| $d$ | Dilution rate (same as supply influx rate to keep volume fixed). |
| Chemostat variables | |
| $\vec{c}_{k}=\left( c_{1,k}, c_{2,k}, \ldots c_{p,k} \right)$ | Chemical environment inside the $k$-th chemostat. $c_{i,k}$ is the concentration of the $i$-th nutrient within the medium of the $k$-th chemostat. All possible $\vec{c}$ constitute the “chemical space”. |
| $m_{\sigma,k}$ | Biomass density of species $\sigma$ in the $k$-th chemostat. |
| Species-specific quantities | |
| $\vec{\alpha}_{\sigma}=(\alpha_{1,\sigma},\alpha_{2,\sigma}\ldots)$ | Resource allocation strategy of species $\sigma$. $\alpha_{j,\sigma}$ is the fraction of internal resources allocated to the $j$-th cellular function by species $\sigma$. |
| $\vec{q}_{\sigma}$ | Intracellular concentrations of growth-related metabolites for species $\sigma$. |
| $g(\vec{c},\vec{q},\vec{\alpha})$ | Growth rate as a function of $\vec{c}$, $\vec{q}$, and $\vec{\alpha}$. |
| $I_{i}(\vec{c},\vec{q},\vec{\alpha})$ | Intake rate per biomass of the $i$-th nutrient as a function of $\vec{c}$, $\vec{q}$, and $\vec{\alpha}$. $I_{i}(\vec{c},\vec{q},\vec{\alpha})$ can be negative to describe cells exporting secondary metabolites. |
| $r$ | Biomass concentration within an average cell, taken to be a constant that is always equal to 100. |
| $\vec{f}(\vec{I}(\vec{c},\vec{q},\vec{\alpha}),\vec{q},\vec{\alpha})$ | Functions defining the changing rate of intracellular metabolite concentrations $\vec{q}$, as a function of $\vec{c}$ , $\vec{q}$, and $\vec{\alpha}$. |
| ${GC}_{\sigma}$ | Growth-rate contour of species $\sigma$. |
| ${FB}_{\sigma}$ | Flux-balance curve of species $\sigma$. |
| $\vec{c}_{\sigma, \text{ss}}$ | The steady-state environment created by one species $\sigma$. |
| $SL_{\sigma}(\vec{c})$ | The supply line for species $\sigma$ in environment $\vec{c}$. |
| $\{\sigma^{*}\}$ | A set of species stably surviving in a chemostat. The set can contain one or more species. |
| $\vec{c}_{\{\sigma^{*}\},\mathrm{ss}}$ | The steady-state environment created by a set of species $\{\sigma^{*}\}$. |

## **Metabolic model and resource allocation strategy**

In modeling population dynamics in a chemostat, multiple assumptions need to be made concerning how cells sense the environment, import nutrients, export metabolites, utilize resources, and grow in biomass. Different assumptions result in different metabolic models. Some metabolic models focus on trade-offs in resource allocation, as the amount of internal resources “owned” by a cell, including proteins and energy, is limited. Cells need to allocate these limited resources into different cellular functions, such as metabolism, gene expression, reproduction, motility, maintenance, etc. We use $\alpha_{j,\sigma}$ to represent the fraction of internal resources allocated to the $j$-th cellular function of species $\sigma$, with $\vec{\alpha}_{\sigma}=(\alpha_{1,\sigma},\alpha_{2,\sigma}\ldots)$ representing the resource allocation strategy of species $\sigma$. For simplicity, we assume each species has a fixed resource allocation strategy.

## **Dynamic equations for a single species in a chemostat**

In a chemostat with nutrient supply $\vec{c}_{\mathrm{supply}}$ , dilution rate $d$ and a single species $\sigma$ with fixed strategy $\vec{\alpha}_{\sigma}$ and intracellular metabolite concentration $\vec{q}_{\sigma}$, the cell biomass density $m_{\sigma}$ and the chemostat nutrient concentrations $\vec{c}$ are generally described by the following equations:

|  | $\frac{dm_{\sigma}}{dt}=m_{\sigma}\cdot\left( g\left( \vec{c},\vec{q}_{\sigma},\vec{\alpha}_{\sigma} \right)-d \right),$ | (S1) |
| --- | --- | --- |
|  | $\frac{d\vec{c}}{dt}=d\cdot\left( \vec{c}_{\mathrm{supply}}-\vec{c} \right)-m_{\sigma}/r\cdot\vec{I}\left( \vec{c},\vec{q}_{\sigma},\vec{\alpha}_{\sigma} \right).$ | (S2) |

In considering the details of cellular metabolism, one may choose to incorporate the dynamics of intracellular metabolites $\vec{q}_{\sigma}$ that originate from nutrient import and influence cell growth. We make the assumption that the biomass concentration $r$, e.g. protein concentration, is constant for cells under all growth conditions. Thus, an increase of total cell mass induces a corresponding increase of total cell volume. (If the volume of the chemostat is $V_{\mathrm{chemostat}}$ and the total volume of cells is $V_{\mathrm{cells}}$, the import flux of the *i*-th nutrient $V_{\mathrm{cells}}\cdot I_{i}$ implies a rate of change of concentration inside cells of $I_{i}$ and a corresponding rate of change of the concentration in the chemostat of $V_{\mathrm{cells}}/V_{\mathrm{chemostat}}\cdot I_{i}=(m\cdot V_{\mathrm{chemostat}}/r)/V_{\mathrm{chemostat}} \cdot I_{i}=m/r\cdot I_{i}$.). $m_{\sigma}$ is the cell mass per element of volume in the chemostat, and $r$ is the cell mass per element of volume within a cell. For a chemostat-to-cell flux of mass *J*, the concentration of the metabolite in the chemostat decrease by $J/V_{\mathrm{chemostat}}$ while the concentration in cell increase by $J/V_{\mathrm{cell}}$. As a result, the metabolites imported into cells are enriched by a factor of $r$, and metabolites secreted by cells are diluted by $1/r$. Also, all intracellular metabolites are diluted by cellular growth, which is generally a slow process compared to metabolic reactions and can be ignored in most cases. Within a cell, the concentration of metabolites is influenced by uptake/secretion rates, and influences the growth rate. Different metabolic models assume different forms for such influences, and we use $\vec{f}\left( \vec{I},\vec{q},\vec{\alpha}_{\sigma} \right)$ to represent the rate of change in $\vec{q}$:

| $\frac{d\vec{q}_{\sigma}}{dt}=\vec{f}\left( \vec{I}\left( \vec{c},\vec{q}_{\sigma},\vec{\alpha}_{\sigma} \right),\vec{q}_{\sigma},\vec{\alpha}_{\sigma} \right).$ | (S3) |
| --- | --- |

Eq. (S2) represents $p$ equations for $p$ types of nutrients, and Eq. (S3) represents $h$ equations for $h$ growth-related intracellular metabolites.

## **Species-specific steady state**

In the steady state of a chemostat, Eqs. (S1)- (S3) will all be equal to zero.

For intracellular metabolites, as $\vec{f}\left( \vec{I}\left( \vec{c},\vec{q}_{\sigma},\vec{\alpha}_{\sigma} \right),\vec{q}_{\sigma},\vec{\alpha}_{\sigma} \right)=0$ as a result of Eq. (S3) being equal to zero, given an environment $\vec{c}$, the steady state of $\vec{q}_{\sigma}$ can be expressed as a function of $\vec{c}$: $\vec{q}_{\sigma}^{*}=\vec{f}^{-1}(\vec{c},\vec{\alpha}_{\sigma})$.

*Growth contour (GC)*

From the perspective of the environment influencing species, at each constant environment, the steady-state growth rate is fully determined by $\vec{c}$: $g^{*}\left( \vec{c,}\vec{q}_{\sigma} \right)=g\left( \vec{c,}\vec{f}^{-1}(\vec{c},\vec{\alpha}_{\sigma}) \right)$. If the biomass of a species is non-zero ($m\neq0$), Eq. (S1) requires $g^{*}=d$. In the $p$-dimensional chemical space, this requirement defines a ($p-1$)-dimensional surface, constituted by all environments $\vec{c}$ that support an equal-to-dilution growth rate. This surface reduces to the zero-growth isoclines in contemporary niche theory when the chemical space is two-dimensional and the growth rate $g$ solely relies on $\vec{c}$ monotonically, but is not necessarily limited by the nutrient dimension or the form of the growth function. For convenience, we name this surface the “growth contour” (GC) for species $\sigma$:

| $GC_{\sigma}:=\left\{ \vec{c} \right\vert g\left( \vec{c},\vec{f}^{-1}\left( \vec{c},\vec{\alpha}_{\sigma} \right),\vec{\alpha}_{\sigma} \right)=d\}.$ | (S4) |
| --- | --- |

Examples of growth contours are shown in Fig 1B and Fig S1.

*Flux-balance curve (FB)*

Eq. (S2) describes how species act on the environment. In steady state, the influx, out-flux, and consumption by species should be balanced for each nutrient, which enables calculation of the biomass density-to-dilution ratio for every $i$: $\frac{m_{\sigma}}{d\cdot r}=\frac{c_{i,\text{ supply}}-c_{i}}{I_{i}\left( \vec{c},\vec{f}^{-1}\left( \vec{c},\vec{\alpha}_{\sigma} \right),\vec{\alpha}_{\sigma} \right)}$. For a $p$-dimensional chemical space, there are $p$ equations for the same value of $\frac{m}{d\cdot r}$. This leads to a one-dimensional curve in the chemical space, which we name the “flux-balance curve” (FB), defined as:

| $FB:=\left\{ \vec{c} \right\vert\frac{c_{i,\text{ supply}}-c_{i}}{I_{i}\left( \vec{c},\vec{f}^{-1}\left( \vec{c},\vec{\alpha}_{\sigma} \right),\vec{\alpha}_{\sigma} \right)}=\frac{m_{\sigma}}{d\cdot r} \mathrm{AND} c_{i}<c_{i,\mathrm{supply}}\}.$ | (S5) |
| --- | --- |

For example, for two nutrients *a* and *b*, the flux balance curve is: $\frac{c_{a,\text{ supply}}-c_{a}}{I_{a}\left( \vec{c},\vec{f}^{-1}\left( \vec{c},\vec{\alpha}_{\sigma} \right),\vec{\alpha}_{\sigma} \right)}-\frac{c_{b,\text{ supply}}-c_{b}}{I_{b}\left( \vec{c},\vec{f}^{-1}\left( \vec{c},\vec{\alpha}_{\sigma} \right),\vec{\alpha}_{\sigma} \right)}=0$, as demonstrated in Fig 1B and Fig S1.

In chemical space, the steady-state environment ($\vec{c}_{\sigma,\text{ss}}$) with non-zero biomass of species $\sigma$ will be located at the intersection of the growth contour and the flux-balance curve. This environment is constructed by the species $\sigma$ via its consumption of nutrients. If $\vec{c}_{\sigma,\text{ss}}$ exists, this species can survive in the chemostat. Otherwise, this species will be washed out by dilution even without competition from other species. For the following discussion, we only consider species that can survive when alone in a chemostat.

*Supply line (SL)*

The flux-balance curve is determined by the supply condition $\vec{c}_{\mathrm{supply}}$. In many cases, it is helpful to derive the supply conditions that enable a species $\sigma$ to construct a steady-state environment $\vec{c}_{\sigma,\text{ss}}$. All possible values of $\vec{c}_{\mathrm{supply}}$ that can produce a given $\vec{c}_{\sigma,\text{ss}}$, form a straight line in the space of supply concentrations, described by:

| $SL:=\left\{ \vec{c}_{\mathrm{supply}} \right\vert\vec{c}_{\mathrm{supply}}=\frac{m_{\sigma}}{d\cdot r}\cdot\vec{I}\left( \vec{c}_{\sigma,\text{ss}},\vec{f}^{-1}\left( \vec{c}_{\sigma,\text{ss}},\vec{\alpha}_{\sigma} \right),\vec{\alpha}_{\sigma} \right)+\vec{c}_{\sigma,\text{ss}}\},$ | (S6) |
| --- | --- |

with varying non-negative values of $m_{\sigma}/d$. Example of supply lines are shown in

Fig S1A. Despite the fact that the supply space and the chemical space are distinct, they share the same units of concentration in each dimension. Therefore, for ease of visualization we typically show supply lines along with other features in the same nutrient space.

## **Control of chemostat**

From the perspective of chemostat-related experiment, this work demonstrates the subtleties of controlling nutrient limitation in chemostats. The capacity of species to shape their own environment, even in a system as simple as a chemostat, presents challenges to controlling which nutrient or nutrients are limiting. By traditional definition, if increasing a certain nutrient leads to an increase of a cell’s growth rate, that nutrient is considered “limiting”. However, growth rate is invariant in a chemostat, being set experimentally by the dilution rate, so inferring nutrient limitation requires special attention. For example, if one sees the same cellular responses under different nutrient supplies, such as the experimental results shown in Fig S2, what can one conclude? Cells may be creating the same chemical environment out of different supply conditions (*cf*. Fig S1A), or alternatively cells may be transducing different chemical environments into the same physiological response through mechanisms such as “ratio sensing” [2]. Our graphical approach combined with direct measurements of steady-state nutrient concentrations in the chemostat can precisely define and help guide the control of nutrient limitation [3]. As described above, changes in supply concentrations shift the flux-balance curve, but do not change the shape of the growth contour. Therefore, by experimentally varying the supply conditions and measuring the chemical environment created by cells, the shape of the growth contour can be obtained. The resulting slope of the growth contour provides information on nutrient limitation even in the absence of detailed knowledge about a cell’s metabolism. For example, in the nutrient *a -* nutrient *b* plane, a near-horizontal growth contour indicates *b*-limited growth while a near-vertical growth contour means *a*-limited growth, and an intermediate slope implies that the two nutrients are co-limiting.

## **Dynamic equations for multiple species in a chemostat**

In nutrient competition models, multiple species ($\sigma=1\ldots n$) each with biomass density $m_{\sigma}$ compete for resources. They have species-specific growth rates $g\left( \vec{c},\vec{q}_{\sigma},\vec{\alpha}_{\sigma} \right)$ and import rates $\vec{I}\left( \vec{c},\vec{q}_{\sigma},\vec{\alpha}_{\sigma} \right)$, yet all experience the same chemical environment $\vec{c}$. Therefore, Eq. (S1) and Eq. (S3) remain the same for each species, while the rate of change of chemostat nutrient concentrations is influenced by the summed action of all species:

|  | $\frac{d\vec{c}}{dt}=d\cdot\left( \vec{c}_{\mathrm{supply}} -\vec{c} \right)-\sum_{\sigma=1}^{n} m_{\sigma}/r\cdot\vec{I}\left( \vec{c},\vec{q}_{\sigma},\vec{\alpha}_{\sigma} \right).$ | (S7) |
| --- | --- | --- |

## **Multiple species steady state**

Multiple species, even if each alone can survive in chemostat, do not generally coexist when competing together. For a system starting with $n$ different species, the stable steady state contains $n^{*}$ ($1\leq n^{*}\leq n$) species with non-zero biomass. We define these $n^{*}$ surviving species as a stable species set $\left\{ \sigma^{*} \right\}$, and mark the steady-state environment created by this set as $\vec{c}_{\{\sigma^{*}\},\text{ss}}$. If $n>1$, according to Eq. (S1), $\vec{c}_{\{\sigma^{*}\},\text{ss}}$ must be located at the common intersection of growth contours formed by every species in $\{\sigma^{*}\}$.

## **Invasion**

Invasion is defined as introducing a small number of invaders (with biomass density $m_{\mathrm{inv}}$) to a steady-state chemostat occupied by a set of local species. At the time of introduction, if the invader can increase in biomass ($\frac{{dm}_{\mathrm{inv}}}{dt}>0$), the invasion is successful; otherwise if the invader decreases in biomass ($\frac{{dm}_{\mathrm{inv}}}{dt}<0$), the invasion is unsuccessful. If the biomass stays constant ($\frac{{dm}_{\mathrm{inv}}}{dt}=0$), the species is neutral with respect to the local species.

In evaluating invasion by a species $\sigma$with strategy $\vec{\alpha}_{\sigma}$ of any environment $\vec{c}$, we make two assumptions:

1. The biomass of the invader is so small that it does not disturb the environment at the time of introduction.

2. There is a separation of timescales such that the concentrations of intracellular metabolites reach equilibrium instantaneously at the time of introduction of the invader, therefore Eq. (S3) is always equal to zero and $\vec{q}_{\sigma}=\vec{f}^{-1}(\vec{c},\vec{\alpha}_{\sigma})$ holds.

Therefore, we define the “invasion growth rate” of a species $\sigma$with strategy $\vec{\alpha}_{\sigma}$ introduced into environment $\vec{c}$ as:

|  | $g_{\mathrm{inv}}\left( \vec{\alpha} \vert\vec{c} \right)=g\left( \vec{c},\vec{f}^{-1}\left( \vec{c},\vec{\alpha} \right),\vec{\alpha} \right).$ | (S8) |
| --- | --- | --- |

*Invasion zone*

By definition, the growth contour of the invader ${GC}_{\text{inv}}$ divides the chemical space into two regions: an “invasion zone” that includes all environments where the invader has an invasion growth rate higher than dilution, and “no-invasion zone” where the invader has an invasion growth rate lower than dilution. If the steady-state environment constructed by local species $\vec{c}_{\{local\},\text{ss}}$ is located within the invasion zone of the invader, $g_{\mathrm{inv}}\left( \vec{\alpha}_{\mathrm{inv}}|\vec{c}_{\{local\},\text{ss}} \right)> d$, therefore $\frac{{dm}_{\mathrm{inv}}}{dt}>0$ by Eq. (S1), and the invasion is successful; otherwise, if $\vec{c}_{\{local\},\text{ss}}$ is located outside of the invasion zone of the invader, $g_{\mathrm{inv}}\left( \vec{\alpha}_{\mathrm{inv}}|\vec{c}_{\{local\},\text{ss}} \right)<d$, and the invasion is unsuccessful. If $\vec{c}_{\{local\},\text{ss}}$ locate exactly on the growth contour, it is neutral.

Two examples of this rule of invasion are presented in Fig 1C-1D.

If the growth rate monotonically increases with the concentration of each nutrient, it can be proven that the invasion zone is always above the growth contour of the invader (an environment $\vec{c}_{+}$ “above” the growth contour ${GC}_{\mathrm{inv}}$ is defined as $\exists\vec{c}_{0} \in{GC}_{\mathrm{inv}}, s.t. c_{i,+}\geq c_{i,0} \forall i$). If the growth rate is not monotonically increasing with nutrient concentrations, identifying the invasion zone requires more model-specific analysis.

## **Fitness landscape**

We quantified the fitness landscapes in the chemostat via the relationship between metabolic strategies $\vec{\alpha}$ and the invasion growth rates of an invader adopting strategy $\vec{\alpha}$ in a given chemical environment $\vec{c}$. Specifically,

|  | $Fitness landscape:=g_{\mathrm{inv}}\left( \vec{\alpha}\vert\vec{c} \right).$ | (S9) |
| --- | --- | --- |

Each environment $\vec{c}$ defines a fitness landscape. A set of species {$\sigma^{*}\}$ constructs a steady-state environment $\vec{c}_{\{\sigma^{*}\},\text{ss}}$ and a corresponding fitness landscape $g_{\mathrm{inv}}\left( \vec{\alpha}|\vec{c}_{\left\{ \sigma\right\},\text{ss}} \right)$. Some examples of fitness landscapes are shown in Figs 2C, 3B-C, 4D, 5D-F, S4D and S6D.

## **Non-invasible/optimal/evolutionarily stable strategies**

A set of species $\left\{ \sigma^{*} \right\}_{\mathrm{opt}}$ is non-invasible, aka optimal or evolutionarily stable, if no other species can invade the steady-state environment constructed by $\left\{ \sigma^{*} \right\}_{\mathrm{opt}}$:

|  | $g_{\mathrm{inv}}\left( \vec{\alpha}_{\sigma}\vert\vec{c}_{\left\{ \sigma^{*} \right\}_{\mathrm{opt}},\mathrm{ss}} \right)<d , \forall\sigma\notin\left\{ \sigma^{*} \right\}_{\mathrm{opt}}.$ | (S10) |
| --- | --- | --- |

Equivalently, Eq. (S10) can be expressed as “a set of species $\left\{ \sigma^{*} \right\}_{\mathrm{opt}}$ construct a fitness landscape which places themselves on the top”, according to Eq. (S9).

The steady state constructed by $\{\sigma^{*}\}$ is influenced by the supply $\vec{c}_{\mathrm{supply}}$ and the dilution rate $d$. For different chemostat parameters, the non-invasible species set $\left\{ \sigma^{*} \right\}_{\mathrm{opt}}$ may be different. In the following steps, we described a generally applicable protocol for obtaining the non-invasible strategies:

*1. Maximal growth rates and maximizing strategies*

In a metabolic model with trade-offs in resource allocation, the maximizing resource allocation strategy $\vec{\alpha}_{\max}$ under a given environment $\vec{c}$ is defined as the strategy that maximizes invasion growth rate:

| $g_{\max}\left( \vec{c} \right):=\max_{\vec{\alpha}} \left( g_{\mathrm{inv}}\left( \vec{\alpha}_{\sigma}\vert\vec{c} \right) \right)$  $\vec{\alpha}_{\max}\left( \vec{c} \right):=\arg\max_{\vec{\alpha}} \left( g_{\mathrm{inv}}\left( \vec{\alpha}_{\sigma}\vert\vec{c} \right) \right).$ | (S11) |
| --- | --- |

*2. Maximal growth contour*

For a given dilution rate $d$, all environments that support a maximal growth rate of $d$ constitute the “maximal growth contour”:

| $GC_{\max}:=\left\{ \vec{c}_{0} \right\vert g_{\max}\left( \vec{c}_{0} \right)=d\}.$ | (S12) |
| --- | --- |

In a multi-species ecosystem, possible steady state can only allocate at the outermost surface of multiple species' growth contours, as highlighted in Fig S3C. More generally, the outermost surface of all species' growth contours forms the boundary of non-invasible strategies.

$GC_{\max}$ is generally formed by many species, with each species adopting the maximizing strategies $\vec{\alpha}_{\max}\left( \vec{c}_{0} \right)$ corresponding to one environment $\vec{c}_{0}$ on the maximal growth contour.

$GC_{\max}$ is outside of the invasion zone for any species $\sigma$. (Otherwise, if a species $\sigma$ could invade an environment $\vec{c}_{0}$ on $GC_{\max}$, $g_{\mathrm{inv}}\left( \vec{\alpha}_{\sigma} | \vec{c}_{0}\in GC_{\max} \right)>d$, this would directly violate the requirement by Eqs. (S11) and (S12) that $\max_{\vec{\alpha}} \left( g_{\mathrm{inv}}\left( \vec{\alpha}_{\sigma}|\vec{c}_{0} \right) \right)=d$.) Therefore, the necessary and sufficient condition for a set of species to be evolutionarily stable, is to construct a steady-state environment on the maximal growth contour:

|  | $\vec{c}_{\left\{ \sigma^{*} \right\}_{\mathrm{opt}},\mathrm{ss}}\in GC_{\max}.$ | (S13) |
| --- | --- | --- |

Therefore, a strategy belonging to the non-invasible set must be a maximizing strategy.

An example of maximal growth contour is shown in Figs 3D, Fig 4B-C, and Fig 5B, C and E.

*3. Non-invasible strategy*

Nevertheless, adopting one of the maximizing strategies along the maximal growth contour does not guarantee that a species will satisfy Eq. (S13) and become non-invasible, as a maximizing strategy for environment $\vec{c}_{1}$ may end up constructing a different environment $\vec{c}_{2}$. To identify a non-invasible species for supply condition $\vec{c}_{\mathrm{supply}}$, the flux-balance condition needs to be considered, with the strategies maximized at each environment. This requirement forms a “maximal flux-balance curve” in the chemical space:

| $FB_{\max}:=\left\{ \vec{c} \right\vert\frac{c_{i,\text{ supply}}-c_{i}}{I_{i}\left( \vec{c},\vec{f}^{-1}\left( \vec{c},\vec{\alpha}_{\max}\left( \vec{c} \right) \right),\vec{\alpha}_{\max}\left( \vec{c} \right) \right)}=\frac{m}{d\cdot r} \mathrm{AND} c_{i}<c_{i,\mathrm{supply}}\}.$ | (S14) |
| --- | --- |

If the intersection of the maximal growth contour and the maximal flux-balance curve exists, it is the evolutionarily stable environment under dilution rate $d$ and supply condition $\vec{c}_{\mathrm{supply}}, \vec{c}_{\mathrm{opt}}.$The maximizing strategy for this environment, $\vec{\alpha}_{\mathrm{opt}}=\vec{\alpha}_{\max}\left( \vec{c}_{\mathrm{opt}} \right)$, constructs the environment $\vec{c}_{\mathrm{opt}}$, and is evolutionarily stable.

*4. Evolutionarily stable coexistence at the discontinuous points of the maximal growth contour* Inversely, for each environment $\vec{c}_{0}$on the maximal growth contour, all supply conditions that enable the maximizing strategy of $\vec{c}_{0}$ to become the non-invasible strategy can be calculated from the supply line according to Eq. (S6):

| $SL(\vec{c}_{0}):=\left\{ \vec{c}_{\mathrm{supply}} \right\vert\vec{c}_{\mathrm{supply}}=x\cdot\vec{I}\left( \vec{c}_{0},\vec{f}^{-1}\left( \vec{c}_{0},\vec{\alpha}_{\max}\left( \vec{c}_{0} \right) \right),\vec{\alpha}_{\max}\left( \vec{c}_{0} \right) \right)+\vec{c}_{0}\},$ | (S15) |
| --- | --- |

for any non-zero value of $x$. Some examples are shown in Fig 3D.

When there are discontinuous points on the maximal growth contour, there can be “gaps” in the nutrient supply space, where no single strategy on the maximal growth contour satisfies Eq. (S14) . Under this condition, more than one strategy is required to co-create an environment on a discontinuous point of the maximal growth contour. Therefore, discontinuous points of the maximal growth contour permit evolutionarily stable coexistence, where $\left\{ \sigma^{*} \right\}_{\mathrm{opt}}$ contains more than one species. Two examples of such discontinuities and coexistence are shown in Fig 4 and Fig 5.

## **Metabolic models**

Different assumptions can be made regarding the metabolic models $\vec{f}(\vec{c},\vec{x},\vec{\alpha})$, $g\left( \vec{c},\vec{x},\vec{\alpha} \right),$ and $\vec{I}\left( \vec{c},\vec{x},\vec{\alpha} \right)$, focusing on various aspects of cellular growth. Different assumptions lead to distinct classes of metabolic models with various results. Nevertheless, our analysis schemes, including the invasion geometry, fitness landscape, and evolutionary stable strategies, are generally applicable for various metabolic models. In this work, we used five metabolic models to illustrate multiple aspects of the species-environment feedback:

*1. Metabolic model with substitutable nutrients*

When two nutrients are mutually substitutable for growth, such as glucose and galactose, the system can be described by metabolic model as shown in Fig 2A. In this metabolic model, we assume an exact trade-off between the allocation of limited resources to import nutrient *a* or nutrient *b*. The fraction of resources allocated to import nutrient *a* is represented by $\alpha_{a}$, thus leaving a fraction $\alpha_{b}=1-\alpha_{a}$ to import nutrient *b*. The import rate of nutrient$i$ is assumed to follow the Monod equation

Aas a function of nutrient concentration, and is proportional to $\alpha_{i}$:

| $I_{i}(\vec{c})=\alpha_{i}\cdot\frac{c_{i}}{c_{i}+K_{i}}$ for $i=a, b.$ | (S16) |
| --- | --- |

Import of the two nutrients contributes additively toward growth rate:

| $g\left( \vec{c} \right)=\gamma\cdot(I_{a}\left( \vec{c} \right)+I_{b}\left( \vec{c} \right)).$ | (S17) |
| --- | --- |

For this model, for simplicity we do not explicitly consider intracellular metabolites. Rather, import directly determines growth.

In this model, a “species” is defined by its value of $\alpha_{a}$.

The **growth contour** satisfies the equation:

| $\alpha_{a}\cdot\frac{c_{a}}{c_{a}+K_{a}}+\left( 1-\alpha_{a} \right)\cdot\frac{c_{b}}{c_{b}+K_{b}}=d/\gamma.$ | (S18) |
| --- | --- |

All growth contours intersect at one point: $\vec{c}$ =[$\frac{K_{a}}{\frac{\gamma}{d}-1},\frac{K_{b}}{\frac{\gamma}{d}-1}$], regardless of the value of $\alpha_{a}$.

The shapes of the two-dimensional growth contours are shown in Fig 1B-D and Fig 2B-D.

Meanwhile, the **flux-balance curve** satisfies:

| $(c_{a,\text{ supply}}-c_{a})\cdot(1-\alpha_{a})\cdot\frac{c_{b}}{c_{b}+K_{b}}=(c_{b,\text{ supply}}-c_{b})\cdot\alpha_{a}\cdot\frac{c_{a}}{c_{a}+K_{a}} ,$ | (S19) |
| --- | --- |

the shape of which is shown in Fig 1B.

The **fitness landscape**, given a steady-state chemical environment $\vec{c}^{*}=\left[ c_{a}^{*},c_{b}^{*} \right]$, is defined as the relationship between instantaneous growth rate and the strategy $\alpha_{a}$:

| $g\left( \alpha_{a}\vert\left[ c_{a}^{*},c_{b}^{*} \right] \right)=\alpha_{a}\cdot\gamma\cdot\left( \frac{c_{a}^{*}}{c_{a}^{*}+K_{a}}-\frac{c_{b}^{*}}{c_{b}^{*}+K_{b}} \right)+\gamma\cdot\frac{c_{b}^{*}}{c_{b}^{*}+K_{b}},$ | (S20) |
| --- | --- |

which is linear function of $\alpha_{a}$ with a slope $\gamma\cdot\left( \frac{c_{a}^{*}}{c_{a}^{*}+K_{a}}-\frac{c_{b}^{*}}{c_{b}^{*}+K_{b}} \right)$. The shapes of the fitness landscape under different $\vec{c}^{*}$ are shown in Fig 2C.

The **maximizing strategy** (Eq. (S11)) under each $\vec{c}^{*}$ can be calculated from Eq. (S20), according to the chemical environment: If $\left( \frac{c_{a}^{*}}{c_{a}^{*}+K_{a}}-\frac{c_{b}^{*}}{c_{b}^{*}+K_{b}} \right)>0$, $\alpha_{a}=1$ maximizes the growth rate; if $\left( \frac{c_{a}^{*}}{c_{a}^{*}+K_{a}}-\frac{c_{b}^{*}}{c_{b}^{*}+K_{b}} \right)<0, \alpha_{a}=0$ maximizes the growth rate; when two species coexist to create the environment $\left[ c_{a}^{*}=\frac{K_{a}}{\frac{\gamma}{d}-1},c_{b}^{*}=\frac{K_{b}}{\frac{\gamma}{d}-1} \right]$, $\left( \frac{c_{a}^{*}}{c_{a}^{*}+K_{a}}-\frac{c_{b}^{*}}{c_{b}^{*}+K_{b}} \right)=0$, then the growth rate in Eq. (S20) becomes constant at $d$ for any strategy (bottom panel in Fig 2C). In this last case, the fitness landscape becomes flat, which allows unlimited coexistence as shown in Fig 2D.

We also tested the model with 10 substitutable nutrients:

| $g\left( \vec{c} \right)=\gamma\cdot\sum_{i=1}^{10} \alpha_{i}\cdot\frac{c_{i}}{c_{i}+K_{i}}.$ | (S21) |
| --- | --- |

In the 10-dimensional chemical space, all growth contours still intersect at the point: $c_{i}^{*}=\frac{K_{i}}{\frac{\gamma}{d}-1}$ for 𝑖$=\left[ 1:10 \right]$. Given a steady-state chemical environment $\vec{c}^{*}$, the growth rate $g$ as a function of $\vec{\alpha}$, $g\left( \vec{\alpha}|\vec{c}^{*} \right)=\sum_{i=1}^{10} \alpha_{i}\cdot\left( \gamma\cdot\frac{c_{i}^{*}}{c_{i}^{*}+K_{i}} \right)$, is also linear with $\alpha_{i}$ in each dimension. Properties of this fitness landscape are similar to those of the two-dimensional model:

1. In a nutrient *k*-enriched environment, namely $\frac{c_{k}^{*}}{c_{k}^{*}+K_{k}}>\frac{c_{i}^{*}}{c_{i}^{*}+K_{i}}, i\neq k$, the maximizing strategy is to allocate the entire budget into enzymes importing the *k*-th nutrient: $\alpha_{k}=1, \alpha_{i}=0 \mathrm{for} i\neq k$. We name the species with such a strategy as the “opportunist” species *k* (Opp *k*). This maximizing strategy leads to the 9 Opp species in Fig 2E-F.

2. At the common intersection point $c_{i}^{*}=\frac{K_{i}}{\frac{\gamma}{d}-1}$, the growth rate is equal to the dilution rate for any strategy.

**Sequential “evolution” in the 10-dimensional chemical space:**

As shown in Fig 2E-F and Fig S3, in this 10-dimensional model, we started with an initial species (Init) with arbitrarily assigned strategy $\vec{\alpha}=[0.17,0.15,0.14,0.12,0.11,0.09,0.08,0.06,0.05,0.03]$, and let it come to steady state in a chemostat with $\vec{c}_{i,\mathrm{supply}}=1, d=1$. Then, in the steady-state environment created by species Init, the “opportunist” species (Opp 1) with the maximal growth rate in that environment was added to the chemostat. Subsequently, in the steady states created by the existing consortia, we identified the fastest growing opportunist species (Opp 2-9) and added them sequentially to the chemostat, until there was no further opportunist strategy with a growth rate higher than the dilution rate (Fig 2E).

In each steady-state environment that appeared during this process, the growth rate of ${10}^{4}$ randomly generated enzyme allocation strategies were tested to assess the “flatness” of the fitness landscape (Fig S3B).

One way to represent competing strategies in a high dimensional nutrient space is to show the outermost part of the growth contours of all competing species. An example for two species competing for two nutrients is shown in Fig S3C. For the substitutable-nutrient model with 10 nutrients, for the *k*-th Opp species ($\alpha_{k}=1, \alpha_{i}=0 \mathrm{for} i\neq k$), the chemical environment that allows its survival but not the survival of the other opportunist species satisfies: $I\left( c_{k} \right)\geq d/\gamma$ and $I\left( c_{i} \right)<d/\gamma$. Therefore, for illustration we used the environment $I\left( c_{k} \right)=d/\gamma, I\left( c_{i} \right)=1/2\cdot d/\gamma$ to represent the outermost growth contour for the *k*-th Opp species. In Fig S3D, these environments are mapped onto a 10-dimensional radar map showing all 10 competing Opp species. The steady-state environment created after adding each species was first mapped to a polygon on the 10 radar-axis, then the centroid of this polygon is plotted with the correct average nutrient level (radius) and indication of dominant nutrients (“angle”) to roughly represent the deviation between the environment in which each of these species is maximizing and the steady-state environment created by that species.

**Parameters**:

Species with the following parameters were used to generate Fig 1 and Fig 2:

| $K_{a}$ | $K_{b}$ | $\gamma$ |
| --- | --- | --- |
| 1.2 | 0.8 | 3 |

The strategy $\alpha_{a}$ varies for different species. In Fig 1C-D and Fig 2B-C, Species *Blue* has $\alpha_{a}=0.2$, species *Red* has $\alpha_{a}=0.6$. Supply conditions are different among the three figures: in Fig 1C, $\vec{c}_{\mathrm{supply}}=[1, 0.5]$; in Fig 1D, $\vec{c}_{\mathrm{supply}}=[0.5, 1]$; in Fig 2B, $\vec{c}_{\mathrm{supply}}=[1,1]$.

All conditions in Fig 2D are the same as in Fig 2B other than that five additional species are added to the system. Their strategies are indicated by the legend at the right.

For the 10-dimensional model shown in Fig 2E-F and Fig S3, $K_{i}=0.5, \gamma=2.$

### *2. Metabolic model with two essential nutrients*

When two nutrients are both essential for growth, such as nitrogen and phosphorus, and both require a substantial allocation of resources for import, the system can be abstractly modeled as shown in Fig 3A. The trade-off and import functions are taken to be the same as in Model 1: *metabolic model with two substitutable nutrients*. However, import of both nutrients is required for cell growth:

| $g\left( \vec{c} \right)=\gamma\cdot\min(I_{a}(\vec{c}),I_{b}(\vec{c})).$ | (S22) |
| --- | --- |

A species is defined by its value of $\alpha_{a}$.

The **growth contour** has the shape of a right angle, with the turning point at $\vec{c}=[K_{a}\cdot\frac{d}{\gamma\cdot\alpha_{a}-d},K_{b}\cdot\frac{d}{\gamma\cdot\alpha_{b}-d}]$, as shown in Fig 3B and Fig S1. The growth contour also determines which nutrient is limiting at steady state: on the vertical line of the growth contour, $c_{a}^{*}=K_{a}\cdot\frac{d}{\gamma\cdot\alpha_{a}-d}$, the growth is limited by nutrient *a*. On the horizontal line of the growth contour, $c_{b}^{*}=K_{b}\cdot\frac{d}{\gamma\cdot\alpha_{b}-d}$, the growth is limited by nutrient *b*.

The **flux balance curve** has the same form as in Eq. (S19).

The **fitness landscape**, given a steady-state chemical environment $\vec{c}^{*}=\left[ c_{a}^{*},c_{b}^{*} \right]$, is defined as the relationship between instantaneous growth rate and the strategy $\alpha_{a}$:

| $g\left( \alpha_{a}\vert\left[ c_{a}^{*},c_{b}^{*} \right] \right)=\gamma\cdot\min(\alpha_{a}\cdot\frac{c_{a}^{*}}{c_{a}^{*}+K_{a}},(1-\alpha_{a})\cdot\frac{c_{b}^{*}}{c_{b}^{*}+K_{b}}),$ | (S23) |
| --- | --- |

which reaches its maximal value

| $g_{\max}=\gamma\cdot\frac{\frac{c_{b}^{*}}{c_{b}^{*}+K_{b}}\cdot\frac{c_{a}^{*}}{c_{a}^{*}+K_{a}}}{\frac{c_{a}^{*}}{c_{a}^{*}+K_{a}}+\frac{c_{b}^{*}}{c_{b}^{*}+K_{b}}}$ | (S24) |
| --- | --- |

under the **maximizing strategy**

| $\alpha_{a,\max}=\frac{\frac{c_{b}^{*}}{c_{b}^{*}+K_{b}}}{\frac{c_{a}^{*}}{c_{a}^{*}+K_{a}}+\frac{c_{b}^{*}}{c_{b}^{*}+K_{b}}}.$ | (S25) |
| --- | --- |

For $\alpha_{a}<\alpha_{a,\max}$, the growth rate is a linearly increasing function $g(\alpha_{a})=\gamma\cdot\alpha_{a}\cdot\frac{c_{a}^{*}}{c_{a}^{*}+K_{a}}$; For $\alpha_{a}>\alpha_{a,\max}$, it is a linearly decreasing function $g(\alpha_{a})=\gamma\cdot(1-\alpha_{a})\cdot\frac{c_{b}^{*}}{c_{b}^{*}+K_{b}}$. These fitness landscapes and corresponding maximizing strategies are shown in Fig 3B, C and E.

The **maximal growth contour** $\boldsymbol{G}\boldsymbol{C}_{\mathbf{max}}$, as defined in Eq. (S12), is

| $\gamma\cdot\frac{\frac{c_{b}}{c_{b}+K_{b}}\cdot\frac{c_{a}}{c_{a}+K_{a}}}{\frac{c_{a}}{c_{a}+K_{a}}+\frac{c_{b}}{c_{b}+K_{b}}}=d$ | (S26) |
| --- | --- |

for this metabolic model.

Inserting $\alpha_{a,\max}$ from Eq. (S25) into Eq. (S19) for flux-balance curve, the **maximal flux-balance curve** $\boldsymbol{F}\boldsymbol{B}_{\text{max}}$, as defined in Eq. (S14) is a linear function:

| $c_{a,\text{ supply}}-c_{a} =c_{b,\text{ supply}}-c_{b}.$ | (S27) |
| --- | --- |

The intersection of the curves specified by Eq. (S26) and Eq. (S27) gives rise to the chemical environment created by the non-invasible metabolic strategy, as shown in Fig 3D.

**Chain of invasion**

As for the substitutable-nutrient model, we performed the sequential “evolution” process for both 2-dimensional and 10-dimensional metabolic models: Starting with an arbitrarily selected initial species (Init) with $\vec{\alpha} =[0.35,0.65]$ (2-dimensional) or $[0.17,0.15,0.14,0.12,0.11,0.09,0.08,0.06,0.05,0.03]$ (10-dimensional), we let Init come to steady state in a chemostat with $\vec{c}_{i,\mathrm{supply}}=1, d=1$. Then, in the steady-state environment created by Init, the species with the maximal growth rate (Eq. (S25)) in that environment was added to the chemostat. Subsequently, in the steady states created by the existing consortia, the species with the maximizing strategy (Eq. (S25)) in that environment was added to the chemostat. This process was repeated 22 times for the 2-dimensional model (Fig 2) and 16 times for the 10-dimensional model (Fig S5). It is observed that the newly added species always outcompetes the previous one, forming a chain of invasion and replacement (Fig 2E and Fig S5D).

In the radar chart in Fig S5E, we qualitatively represent some of the 10-dimensional growth contours formed during the chain of invasion. According to Eq. (S25) a maximizing strategy $\vec{\alpha}$intersects the optimal growth contour at the environment:$c_{i}=\frac{d}{\gamma\cdot\alpha_{i}-d}\cdot K_{i}$. This environment favors the strategy $\vec{\alpha}$ above all others, and therefore this environment is representative of the growth contour of strategy $\vec{\alpha}$. The radar chart in Fig. S5E shows these representative environments for species 1, 8, and 15. The steady-state environment created by each species was first mapped to a polygon on the 10 radar-axes, then the centroid of this polygon was plotted as a dot to roughly represent the deviation between the environment in which each of these species is maximizing and the steady-state environment created by that species.

**Parameters:** A species with the following parameters was used to generate Fig S1, focusing on how supply conditions and dilution rate influence nutrient limitation:

| $K_{a}$ | $K_{b}$ | $\gamma$ | $\alpha_{a}$ |
| --- | --- | --- | --- |
| 0.7 | 1.3 | 10 | 0.3 |

In Fig S1A, to demonstrate how species construct the same environment out of different supply conditions, the chemostat dilution rate was set to $d=1$, and three supply conditions were used: $\vec{c}_{\mathrm{supply}}=[0.6, 0.3546]$ (purple), $\vec{c}_{\mathrm{supply}}=[ 0.8, 0.5273]$ (cyan), and $\vec{c}_{\mathrm{supply}}=[1,0.7]$ (blue).

In Fig S1B, to demonstrate how dilution rates may switch the limiting nutrient, we used the supply condition $\vec{c}_{\mathrm{supply}}=[1,0.7]$, and three dilution rates: 0.5 (yellow), 1 (red), and 1.6 (deep red).

Species with following parameters were used to generate Fig 3B-E:

| $K_{a}$ | $K_{b}$ | $\gamma$ |
| --- | --- | --- |
| 0.5 | 0.5 | 10 |

The strategy $\alpha_{a}$ varies for different species. In Fig 3B, Species *Blue* has $\alpha_{a}=0.35$, species *Red* has $\alpha_{a}=0.65$. In Fig 3C, we started with species *Blue* and species *Red*. We then generated the fitness landscape for each species at the steady-state environment it constructed, then chose the strategy that maximized invasion growth rate for this fitness landscape to generate a new species, and iterated this process five times. The species *Black* has $\alpha_{a}=0.5$.

### *3. Metabolic model with substitutable nutrients that require assimilation*

In cells, the assimilation of imported raw material, such sugars, into biomass such as proteins, takes multiple steps and enzymes and consumes a considerable amount of energy. When the resources allocated to nutrient assimilation are considered, a cell’s strategy becomes more complex. A mathematical model involving three substitutable nutrients *a, b, c* that need assimilation is shown in Fig S4A, with $\alpha_{i1}$ represents the fraction of resources allocated to importing nutrient *i* into internal metabolite, and $\alpha_{i2}$ represents the fraction of resources allocated to assimilate the internal *i* into biomass. In this model, the import rate has a similar form to the previous two models,

| $I_{i}\left( c_{i} \right)=V\cdot\alpha_{i1}\cdot\frac{c_{i}}{c_{i}+K_{i}}.$ | (S28) |
| --- | --- |

The internal metabolite concentration $c_{i,\mathrm{internal}}$ has an influx of $r\cdot I_{i}\left( c_{i} \right)$, meanwhile, it is diluted by cell growth at the rate $g$. We assume all nutrients are substitutable therefore the internal pools contribute via summation to growth, and are converted into biomass at a rate $k\cdot\alpha_{i2}\cdot c_{i,\mathrm{internal}}$:

| $\frac{dc_{i,\mathrm{internal}}}{dt}=I_{i}\left( c_{i} \right)-g(\vec{c}_{\mathrm{internal}})\cdot c_{i,\mathrm{internal}}-k\cdot\alpha_{i2}\cdot c_{i,\mathrm{internal}}.$ | (S29) |
| --- | --- |

Therefore, the mass converted into biomass per unit time per unit volume is: $\sum_{i} (k\cdot\alpha_{i2}\cdot c_{i,\mathrm{internal}})$, and the growth rate defined as the relative gain of total biomass *M* is:

| $g(\vec{c}_{\mathrm{internal}})=\frac{\frac{dM}{dt}}{M}=\frac{k}{r}\cdot\sum_{i} \left( \alpha_{i2}\cdot c_{i,\mathrm{internal}} \right).$ | (S30) |
| --- | --- |

The growth contour in 3-dimensional chemical space satisfies

| $d\cdot\frac{r}{k} =\alpha_{a2}\cdot\alpha_{a1}\cdot\frac{V\cdot\frac{c_{a}}{c_{a}+K_{a}}}{d+k\cdot\alpha_{a2}}+\alpha_{b2}\cdot\alpha_{b1}\cdot\frac{V\cdot\frac{c_{b}}{c_{b}+K_{b}}}{d+k\cdot\alpha_{b2}}+\alpha_{c2}\cdot\alpha_{c1}\cdot\frac{V\cdot\frac{c_{c}}{K_{c}+c_{c}}}{d+k\cdot\alpha_{c2}},$ | (S31) |
| --- | --- |

As shown in Fig S4C.

**Rock-paper-scissors fitness landscape:**

In the metabolic model shown in Fig S4A, three substitutable nutrients, *a*, *b*, and *c*, contribute additively to cell growth. In this three-dimensional chemical space, the growth contour for each species is a two-dimensional surface (Fig S4C). In addition to requiring enzymes to import the raw forms of these nutrients as in the model of Fig 2A, enzymes are also required to convert the imported raw materials into biomass. In this model, a six-element $\vec{\alpha}$ is required to describe the metabolic strategy, and there is the possibility of “mismatches” in the fraction of internal resources allocated to import and to convert the same nutrient. Such mismatches can produce a “rock-paper-scissors” invasion loop (Fig S4B): species 1 (*Red*, $\alpha_{c1}$ > $\alpha_{a1}$ > $\alpha_{b1}$, $\alpha_{b2}\sim\alpha_{a2}>\alpha_{c2}$) is good at importing nutrient *c* but favors an environment with a high concentration of *a* (see Fig S4B). Therefore, in the environment created by species 1, nutrient *c* is low while *b* is high. This environment is inviting for species 2 (*Green*) which is good at importing *a* but prefers *b*, but not for species 3 (*Blue*) which is good at importing *b* but prefers *c*. Such a mismatch between “imported nutrients” and “preferred nutrients” allows for the rock-paper-scissors loop of invasion. Such a loop of invasions leads to oscillatory population dynamics (Fig S4D, upper panel), with an ever-changing fitness landscape.

**Stochastic simulation:**

In order to assess the stability of the rock-paper-scissors oscillatory dynamics under stochastic fluctuations, white noise was superimposed to the dynamical equations Eq. (S28-S29), following the simplest form of an Ito process:

| $dX=A\left( X \right)dt+\xi\cdot\sqrt{dt},$ | (S32) |
| --- | --- |

where *X* represents all variables. $A(X)$ are the ordinary differential equations for the chemostat. $\xi$ is the noise term which follows a normal distribution with mean 0 and variance $\sigma^{2}$.

The stochastic simulation shows that if species are allowed to recover by stochasticity after dropping to zero biomass, this system exhibits sustained oscillatory dynamics (Fig S4E). By contrast, if a species is considered to become extinct after dropping to zero biomass, the system randomly ends up in a single-species state after an extinction event. Coexistence of two species is not possible (Fig S4F).

**Parameters:**

In generating Fig S4B-C, the chemostat parameters were: $\vec{c}_{\mathrm{supply}}=[1,1,1]$, and $d=1$, and the species parameters were:

| $V$ | $K_{i} (i=a,b,c)$ | $k$ |
| --- | --- | --- |
| 1000 | 0.5 | 11 |

The three species allocate their resources differently:

| Strategies | $\alpha_{a1}$ | $\alpha_{a2}$ | $\alpha_{b1}$ | $\alpha_{b2}$ | $\alpha_{c1}$ | $\alpha_{c2}$ |
| --- | --- | --- | --- | --- | --- | --- |
| *Red* | 0.15 | 0.2 | 0.1 | 0.25 | 0.26 | 0.04 |
| *Green* | 0.26 | 0.04 | 0.15 | 0.2 | 0.1 | 0.25 |
| *Blue* | 0.1 | 0.25 | 0.26 | 0.04 | 0.15 | 0.2 |

In stochastic simulation, $\sigma=0.02$ for all varibles.

### *4. Metabolic model with essential nutrients that can be interconverted*

If two nutrients are both essential for growth, and a cell is able to convert one nutrient into another albeit at a certain cost, as shown in Fig 4A, metabolic trade-offs involve the following four elements of the allocation strategy $\vec{\alpha}$ :

$\alpha_{a}$: Fraction of resources allocated to import nutrient *a*.

$\alpha_{b}$: Fraction of resources allocated to import nutrient *b*.

$\alpha_{ab}$: Fraction of resources allocated to convert internal *b* into *a*.

$\alpha_{ba}$: Fraction of resources allocated to convert internal *a* into *b*.

To implement trade-offs, the sum of elements of $\vec{\alpha}=(\alpha_{a},\alpha_{b},\alpha_{ab},\alpha_{ba})$ is taken to be equal to 1.

In this metabolic model, cells internalize nutrient *a* and nutrient *b* from the chemostat to supply internal concentration of nutrients, $c_{a,\mathrm{internal}}$ and $c_{b,\mathrm{internal}}$. Meanwhile, the internal nutrients can be converted into each other. Nutrients also diffuse in and out of the cell passively with rate $\beta$. Cell growth requires both internal nutrients, and depletes them in a fixed proportion.

In this model, the growth rate of a cell is taken to be:

| $g(\vec{c}_{\mathrm{internal}})=\frac{\gamma}{\frac{K_{a}}{c_{a,\mathrm{internal}}}+\frac{K_{b}}{c_{b,\mathrm{internal}}}}.$ | (S33) |
| --- | --- |

The net import rate, including passive diffusion, is:

| $I_{i}=\left( \alpha_{i}+\beta\right)\cdot c_{i}-\beta\cdot c_{i,\mathrm{internal}}$, $i=a, b.$ | (S34) |
| --- | --- |

Therefore, the dynamical equations for the internal nutrients are:

| $\frac{dc_{a,\mathrm{internal}}}{dt}=I_{a}+\alpha_{ab}\cdot c_{b,\mathrm{internal}}-\alpha_{ba}\cdot c_{a,\mathrm{internal}}-g/K_{a},$ | (S35) |
| --- | --- |
| $\frac{dc_{b,\mathrm{internal}}}{dt}=I_{b}+\alpha_{ba}\cdot c_{a,\mathrm{internal}}-\alpha_{ab}\cdot c_{b,\mathrm{internal}}-g/K_{b}.$ | (S36) |

A species is defined by its value of $\vec{\alpha}$.

This metabolic model was used to demonstrate how to obtain locally optimal strategies and cartels, as shown in Fig 4.

The growth contour in this metabolic model obeys:

| $\frac{\gamma}{d\cdot\beta\cdot\left( \alpha_{ab}+ \alpha_{ba}+ \beta\right)}=\frac{K_{a}}{c_{a}\cdot\beta^{2}+ \alpha_{a}\cdot\alpha_{ab}\cdot c_{a}+ \alpha_{b}\cdot\alpha_{ab}\cdot c_{b}- \alpha_{ab}\cdot K_{a}\cdot d - \alpha_{ab}\cdot K_{b}\cdot d + \alpha_{a}\cdot c_{a}\cdot\beta+ \alpha_{ab}\cdot c_{a}\cdot\beta+ \alpha_{ab}\cdot c_{b}\cdot\beta- K_{a}\cdot d\cdot\beta}+\frac{K_{b}}{c_{b}\cdot\beta^{2}+ \alpha_{a}\cdot\alpha_{ba}\cdot c_{a}+ \alpha_{b}\cdot\alpha_{ba}\cdot c_{b}- \alpha_{ba}\cdot K_{a}\cdot d - \alpha_{ba}\cdot K_{b}\cdot d + \alpha_{ba}\cdot c_{a}\cdot\beta+ \alpha_{b}\cdot c_{b}\cdot\beta+ \alpha_{ba}\cdot c_{b}\cdot\beta- K_{b}\cdot d\cdot\beta}$ | (S37) |
| --- | --- |

In generating Fig 4B, we searched for the maximizing strategies in chemical space, and classified them by their nonzero values. Maximal growth contours for four dilution rates: 0.1, 0.2, 0.3, 0.4 are shown from black to gray and white colors.

Three types of maximizing strategies appeared in the search (Fig S7). The "importer" type has $\alpha_{ab}=0$ and $\alpha_{ba}=0$. There are two “converter” types: one only converts *a* into *b* ($\alpha_{ab}=0,\alpha_{b}=0$), and another converts *b* into *a* ($\alpha_{ba}=0 ,\alpha_{a}=0$).

In generating Fig 4C, the chemostat parameters were set to $\vec{c}_{\mathrm{supply}}=\left[ 0.5,1 \right]$, and $d=0.2$. The maximal growth contours for $d=0.2$ were drawn, along with maximizing strategies along the contour shown as squares with colors corresponding to their sub-classes. At the discontinuous point of the maximal growth contour where the “converter” and the “importer” converge, the distinct two maximizing strategies are denoted species *Red* and species *Blue*. In generating the competition dynamics in inset, additional to the species *Red* and species *Blue*, ten other maximizing strategies along the maximal growth contours were chosen.

**Parameters:**

The parameter values used to generate the plots in Fig 4B-D were:

| $\gamma$ | $K_{i} (i=a,b)$ | $\beta$ |
| --- | --- | --- |
| 1 | 1 | 0.2 |

### *5. Metabolic model with multiple energy generating steps*

Cell growth is also tightly coupled with energy production. For example, with a single carbon supply as the energy source, cells employ multistep reactions to generate multiple ATP molecules. Each step requires dedicated enzymes. The reaction intermediates, such as acetate, usually have dual roles: on the one hand, they positively contribute to ATP production via downstream reactions; on the other hand, they negatively contribute to ATP production by hampering upstream reactions. To deal with the negative effects of intermediates, cells may transport them out into the environment, generally with some metabolic cost for transporters. On the other hand, cells can also uptake such intermediates and use them as an energy source.

We abstract such a process by the model shown in Fig 5A. A single chemical energy source S is supplied into the chemostat, which can be converted into intermediate I by cells. Four reactions are possible in this model, each mediated by a specific enzyme:

1. Import the resource S into the cell and convert it into internal intermediate I_int_ to extract energy (e.g. ATP). The fraction of the model enzyme budget allocated to this reaction is $\alpha_{ATP1}$. We assume the reaction is reversible, with the concentration of S contributing positively to the reaction rate while the concentration of I_int_ contributes negatively:

| $J_{1}=\alpha_{ATP1}\cdot V_{1}\cdot\frac{\left[ S \right]-\frac{\left[ I_{\mathrm{int}} \right]}{K_{3}}}{K_{1}+\left[ S \right]+\frac{\left[ I_{\mathrm{int}} \right]}{K_{5}}}.$ | (S38) |
| --- | --- |

2. Process I_int_ via a downstream reaction to obtain more energy. The fraction of enzymes being allocated to this reaction is $\alpha_{ATP2}$. For this model system, it does not qualitatively influence the final results whether this reaction is product inhibited, so we neglect product inhibition. For simplicity, we assume this reaction has Michaelis–Menten form:

| $J_{2}=\alpha_{ATP2}\cdot V_{2}\cdot\frac{\left[ I_{\mathrm{int}} \right]}{K_{2}+\left[ I_{\mathrm{int}} \right]}.$ | (S39) |
| --- | --- |

3. Export the internal intermediate out into the environment by diffusion, with a fraction of proteins $\alpha_{\exp}$ allocated to channels that allow the excretion of the intermediate into the environment to become external intermediate I_ext._

| $J_{3}=\alpha_{\exp}\cdot k\cdot\left( \left[ I_{\mathrm{int}} \right]-\left[ I_{\mathrm{ext}} \right] \right).$ | (S40) |
| --- | --- |

4. Import the external intermediate into cells, with a fraction of proteins $\alpha_{\mathrm{imp}}$ allocated to the import process. To reflect the property of the internal intermediate in inhibiting this transport reaction, the rate for this process is also product-inhibited:

| $J_{4}=\alpha_{\mathrm{imp}}\cdot V_{4}\cdot\frac{\left[ I_{\mathrm{ext}} \right]-\frac{\left[ I_{\mathrm{int}} \right]}{K_{6}}}{K_{4}+\left[ I_{\mathrm{ext}} \right]+\frac{\left[ I_{\mathrm{int}} \right]}{K_{7}}}.$ | (S41) |
| --- | --- |

Under this model, the rate of change of the concentration of the energy source S in the chemostat is:

| $\frac{d\left[ S \right]}{dt}=d\cdot\left( \left[ S_{\mathrm{supply}} \right]-\left[ S \right] \right)-\frac{m}{r}\cdot J_{1}.$ | (S42) |
| --- | --- |

The rate of change of the external intermediate concentration in the chemostat is:

| $\frac{d\left[ I_{\mathrm{ext}} \right]}{dt}=d\cdot\left( -\left[ I_{\mathrm{ext}} \right] \right)-m/r\cdot\left( J_{4}-J_{3} \right).$ | (S43) |
| --- | --- |

The concentration of the intracellular metabolite I_int_ follows the equation:

| $\frac{d\left[ I_{\mathrm{int}} \right]}{dt}=J_{1}-J_{2}-J_{3}+J_{4}.$ | (S44) |
| --- | --- |

The growth rate is a weighted sum of the ATP produced by $J_{1}$ and $J_{2}$:

| $g=n_{ATP1}\cdot J_{1}+n_{ATP2}\cdot J_{2}.$ | (S45) |
| --- | --- |

Equations for the growth contours and maximizing strategies are long. They are presented in the program package.

**Parameters:**

In generating plots in Fig 5B-F, the species parameters were:

| $V_{1}$ | $V_{2}$ | $k$ | $V_{4}$ | $K_{1}$ | $K_{2}$ | $K_{3}$ | $K_{4}$ | $K_{5}$ | $K_{6}$ | $K_{7}$ | $n_{ATP1}$ | $n_{ATP2}$ |
| --- | --- | --- | --- | --- | --- | --- | --- | --- | --- | --- | --- | --- |
| 5 | 1 | 8 | 10 | 0.5 | 0.5 | 0.5 | 0.1 | 0.5 | 15 | 10 | 1 | 1 |

Maximal growth contours for dilution rates 0.2, 0.4, and 0.6 are shown in Fig 5B.

For Fig 5C-D, the chemostat parameters are: $S_{\mathrm{supply}}=1, d=0.4$.

For Fig 5E-F, the chemostat parameters are: $S_{\mathrm{supply}}=1.8, d=0.6$.

A summary of maximizing strategies in chemical space is shown in Fig S7.

## **Dynamic equations for multiple species in a chain of chemostats**

Real ecosystems seldom exist in isolation. We modeled interconnected ecosystems via a chain of chemostats labeled $k=1$ to $k_{\mathrm{tot}}$ (Fig S6A). Each chemostat exchanges medium and cells at leakage rate$l$ with its two neighboring chemostats (if $k=1$ or $k=k_{\mathrm{tot}}$, there is only one neighbor). The chemostat parameters $\vec{c}_{\mathrm{supply}}$ and $d$ are taken to be identical for all chemostats.

For the $k$-th chemostat, the dynamical equations for the biomass density of species $\sigma$ and the concentration of the $i$-th nutrient are:

|  | $\frac{dm_{\sigma,k}}{dt}=m_{\sigma,k}\cdot\left( g_{\sigma}\left( \vec{c}_{k} \right)-d \right)+l\cdot(m_{\sigma,k-1}+m_{\sigma,k+1}-2\cdot m_{\sigma,k}),$ | (S46) |
| --- | --- | --- |
|  | $\frac{dc_{i,k}}{dt}=d\cdot\left( c_{i,\text{ supply}}-c_{i,k} \right)-\sum_{\sigma=1}^{n} m_{\sigma,k}\cdot I_{i,\sigma}\left( \vec{c}_{k} \right)+l\cdot\left( c_{i,k+1}+c_{i,k-1}-2\cdot c_{i,k} \right).$ | (S47) |

A steady-state solution to these equations is shown in Fig S6, using the same growth and import models and parameters as in Fig 3, with the leakage rate set to be $l=1$.

**REFERENCES**

1. Li SH-J, Li Z, Park JO, King CG, Rabinowitz JD, Wingreen NS, et al. Escherichia coli translation strategies differ across carbon, nitrogen and phosphorus limitation conditions. Nature microbiology. 2018;3(8):939.

2. Escalante-Chong R, Savir Y, Carroll SM, Ingraham JB, Wang J, Marx CJ, et al. Galactose metabolic genes in yeast respond to a ratio of galactose and glucose. Proc Natl Acad Sci U S A. 2015;112(5):1636-41. doi: 10.1073/pnas.1418058112. PubMed PMID: 25605920; PubMed Central PMCID: PMCPMC4321281.

3. Boer VM, Crutchfield CA, Bradley PH, Botstein D, Rabinowitz JD. Growth-limiting intracellular metabolites in yeast growing under diverse nutrient limitations. Mol Biol Cell. 2010;21(1):198-211. doi: 10.1091/mbc.E09-07-0597. PubMed PMID: 19889834; PubMed Central PMCID: PMCPMC2801714.
