# Supplementary figures and images for: Modeling microbial metabolic trade-offs in a chemostat
Source: PLoS Comput Biol. 2020 Aug 28;16(8):e1008156. doi: 10.1371/journal.pcbi.1008156 (PMC7482850; doi:10.1371/journal.pcbi.1008156)

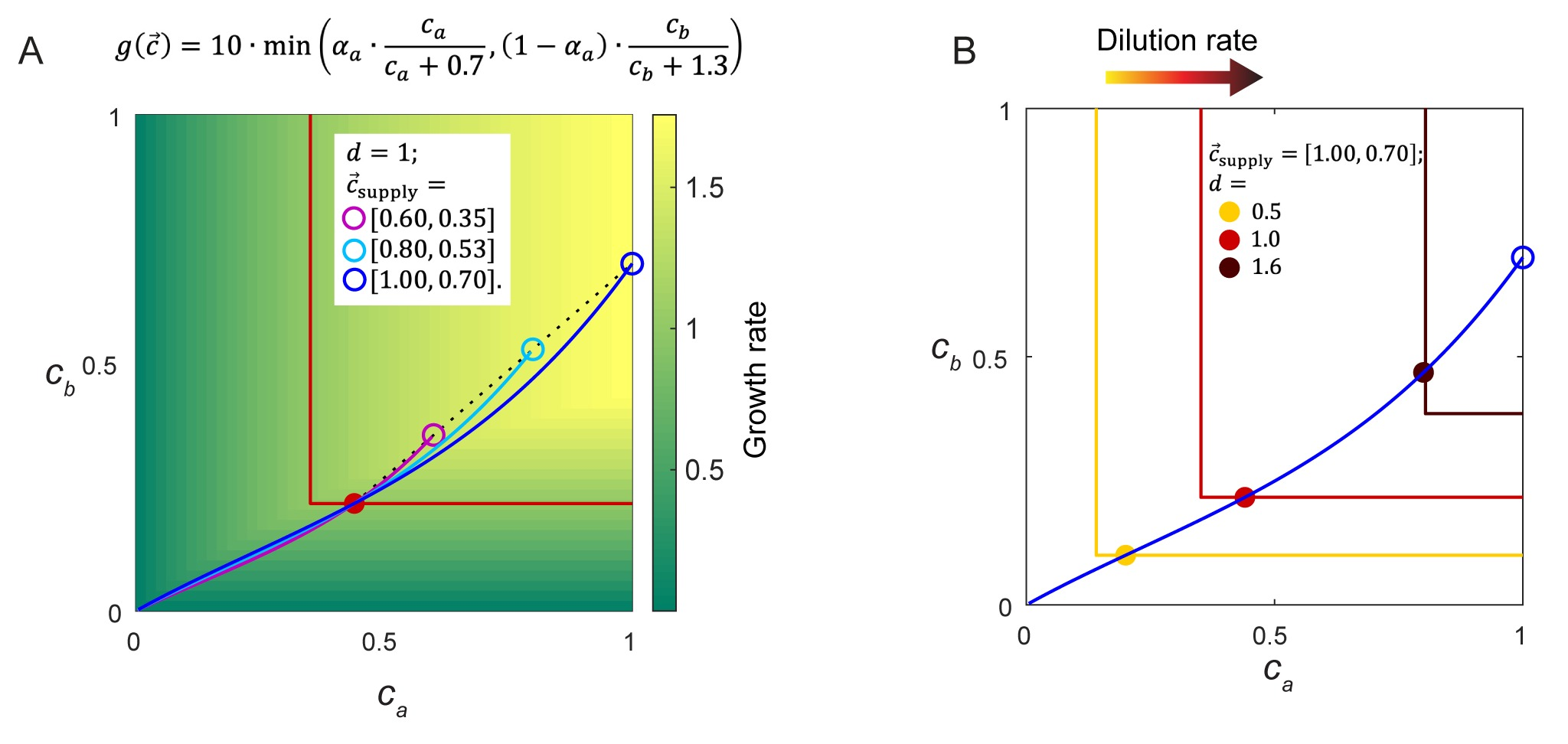

Supplement: S1 Fig — A. Various supply concentrations can lead to the same steady-state chemical environment. Background color indicates the growth rate of cells as a function of nutrient concentrations ca and cb, with the growth contour shown by the red curve. The supply line for the steady-state environment (purple dot) is shown as a dotted black line. Different supply concentrations (ca,supply and cb,supply) along the supply line are marked by purple, cyan, and blue circles, with the corresponding flux-balance curves shown in the same colors. B. Dilution rate can flip nutrient limitation. The external supply condition is marked by a blue circle, and the flux-balance curve for this supply is shown in the same color. Three growth contours with increasing dilution rates are shown from yellow to deep red, and the corresponding steady-state environments are shown as colored dots. (TIF) [file pcbi.1008156.s002.tif]

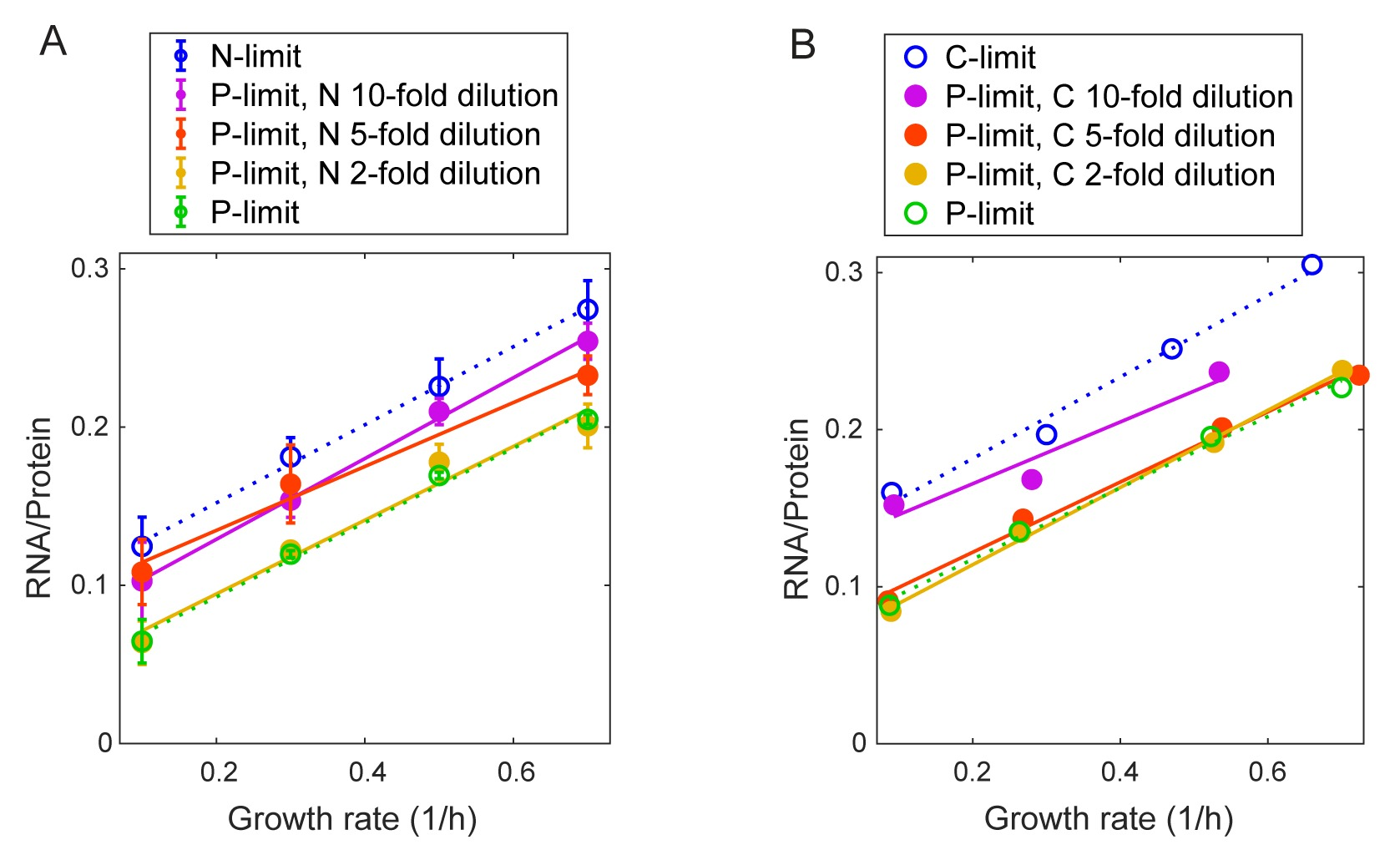

Supplement: S2 Fig — A. The relationship between ribosome abundance represented by RNA/Protein ratio (y-axis) and growth rate (x-axis) of E. coli cultured in chemostats from phosphorus limitation (P-limited, green open circles and dotted line) to nitrogen limitation (N-limited, blue open circles and dotted line). Starting from the P-limited condition, data for decreasing the supply concentration of nitrogen by 2, 5, and 10-fold are shown as solid dots and corresponding best-fit lines. Each measurement was repeated three times and standard errors are shown by bars. C. Same as (B), but for phosphorus and carbon limitation instead of phosphorus and nitrogen limitation. Starting from the P-limited condition, data for decreasing the supply concentration of carbon by 2, 5, and 10-fold are shown as solid dots and corresponding best-fit lines. (TIF) [file pcbi.1008156.s003.tif]

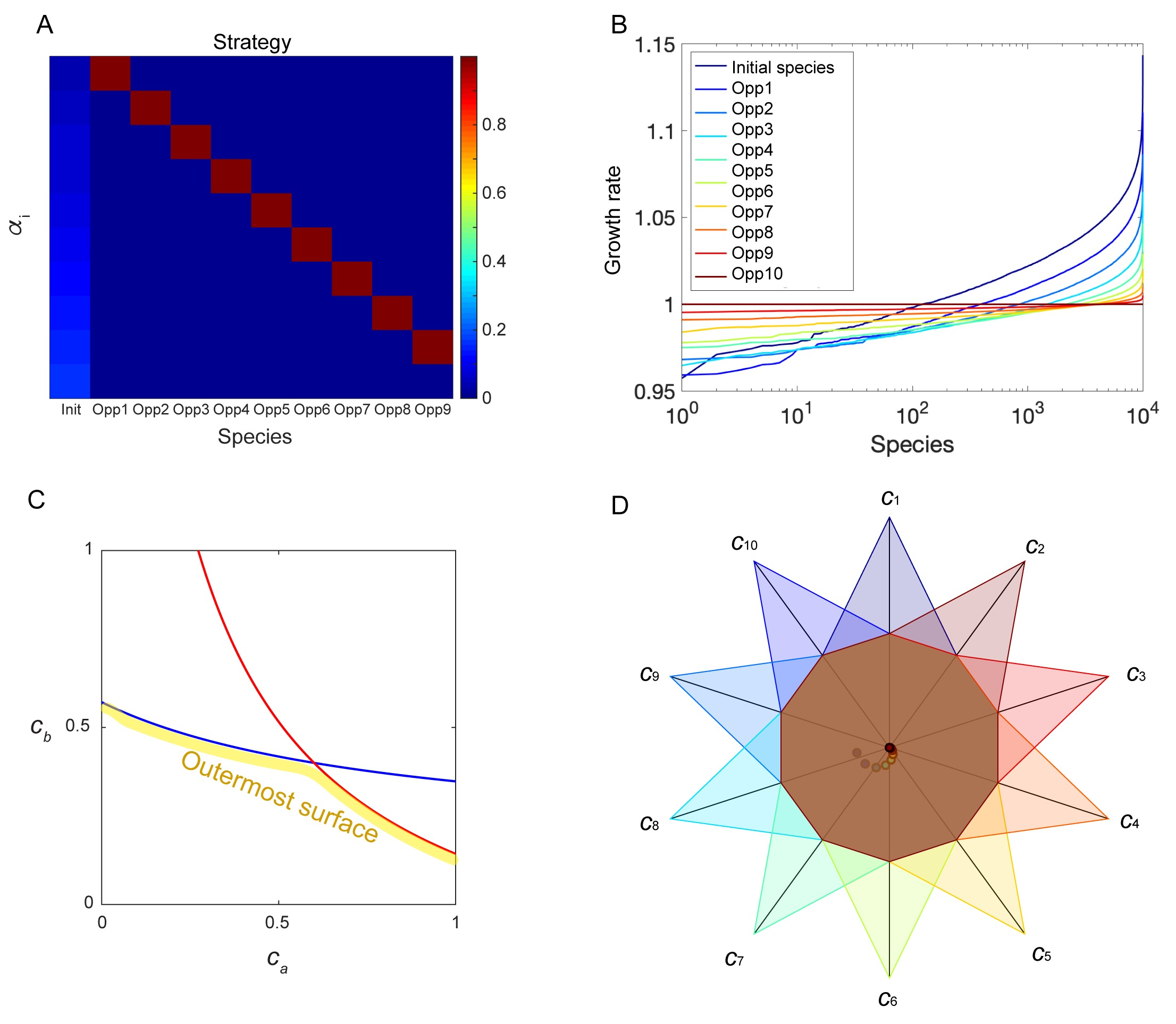

Supplement: S3 Fig — A. The enzyme allocation strategies of the initial species (Init) and the 9 opportunist species (Opp 1–9) that appear in succession in Fig 2E. B. The instantaneous growth rates of 104 randomly generated enzyme allocation strategies, under the steady-state chemical environments created by consecutively adding the species shown in (A) into the existing consortia. C. Example of the “outermost” surface formed by multiple growth contours, as highlighted in yellow. D. Rescaled radar plot for the “most favorable environment” for each strategy that appears in (A). Dots represent the centroids of polygons representing the chemical environments created after adding each species (see S1 Appendix “Metabolic model with substitutable nutrients” for details). (TIF) [file pcbi.1008156.s004.tif]

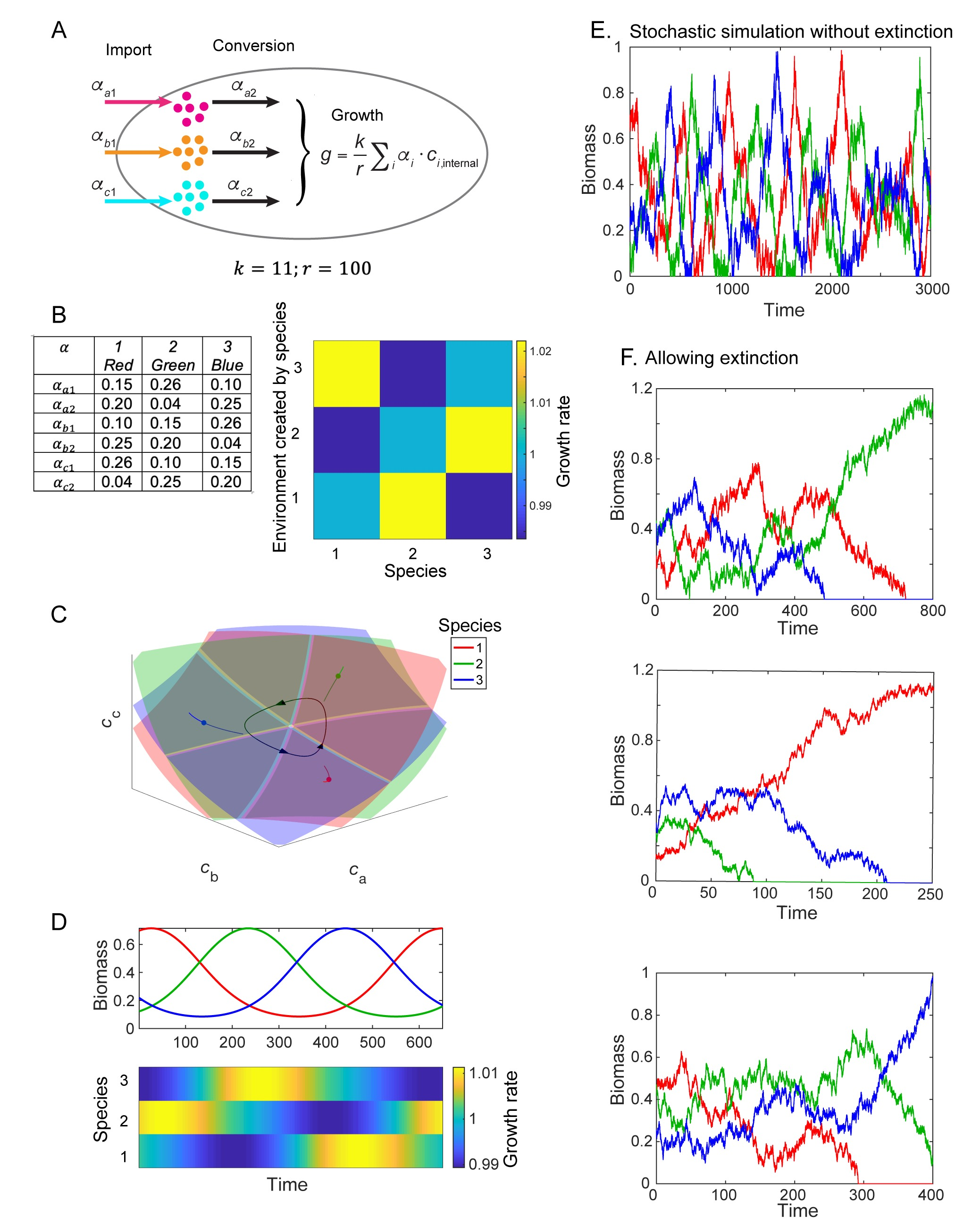

Supplement: S4 Fig — Example of a metabolic model with a trade-off in allocation of internal resources for import and assimilation of three substitutable nutrients, with all three nutrients contributing additively to growth. Species 1 (Red), species 2 (Blue), and species 3 (Green) allocate resources differently (see S1 Appendix). B. The fitness of Species 1, 2, and 3 in the steady-state environment constructed by species 1, 2, and 3. C. Growth contours (surfaces), flux-balance curves (lines), and steady-state nutrient concentrations (dots) for the three species in a three-dimensional chemical space. Black curves with arrows show the system’s limit-cycle trajectory. D. The upper panel shows the time course of species biomass in the chemostat for the limit cycle in (C). The bottom panel shows how the fitness landscape changes with time over one period of the oscillation. E. Stochastic simulation of the model shown in Fig 3, where species are never allowed to drop to zero biomass. F. Same as (E), except that species are considered to become extinct after dropping to zero biomass. (TIF) [file pcbi.1008156.s005.tif]

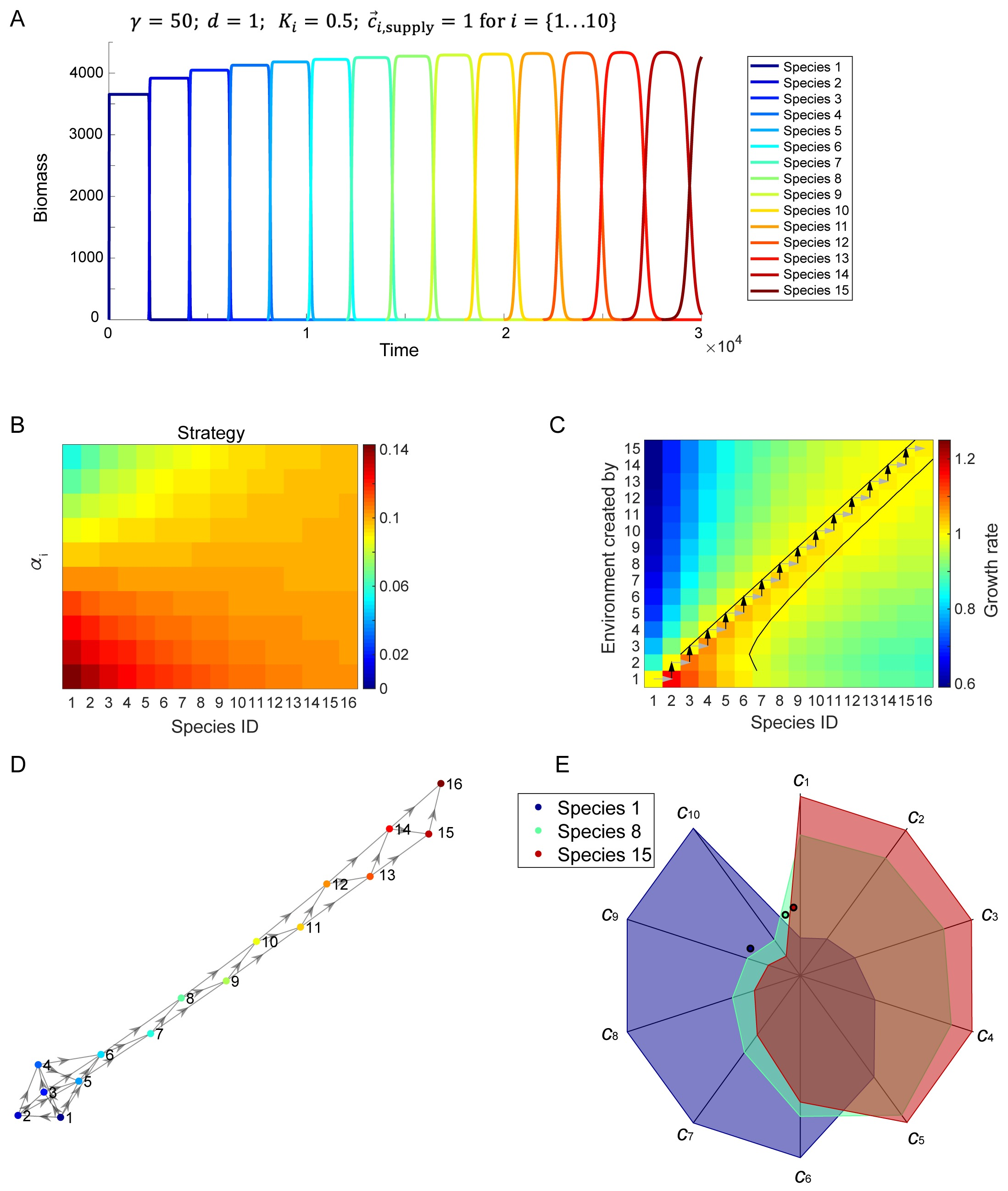

Supplement: S5 Fig — A. Population dynamics of the metabolic model with 10 essential nutrients, where each newly introduced species has the fastest-growing strategy in the steady-state environment created by the existing consortium. B. The enzyme allocation strategies of the species that appear in succession in (A). C. The instantaneous growth rates of the strategies that appear in (A) under the steady-state environments created by the existing consortia. Gray arrows indicate addition of the new species with the fastest-growing strategy, and black arrows indicate the change of the steady-state chemical environment induced by adding this new species. Black contours indicate growth rate equal to dilution rate. D. The chain of invasion. Each colored dot represents one species in (A). An arrow from species i to species j indicates successful invasion of j into the environment created by i alone. E. Rescaled radar plot for the “most favorable environment” for each strategy that appears in (A). Dots represent the center of the chemical environment created by each species. (TIF) [file pcbi.1008156.s006.tif]

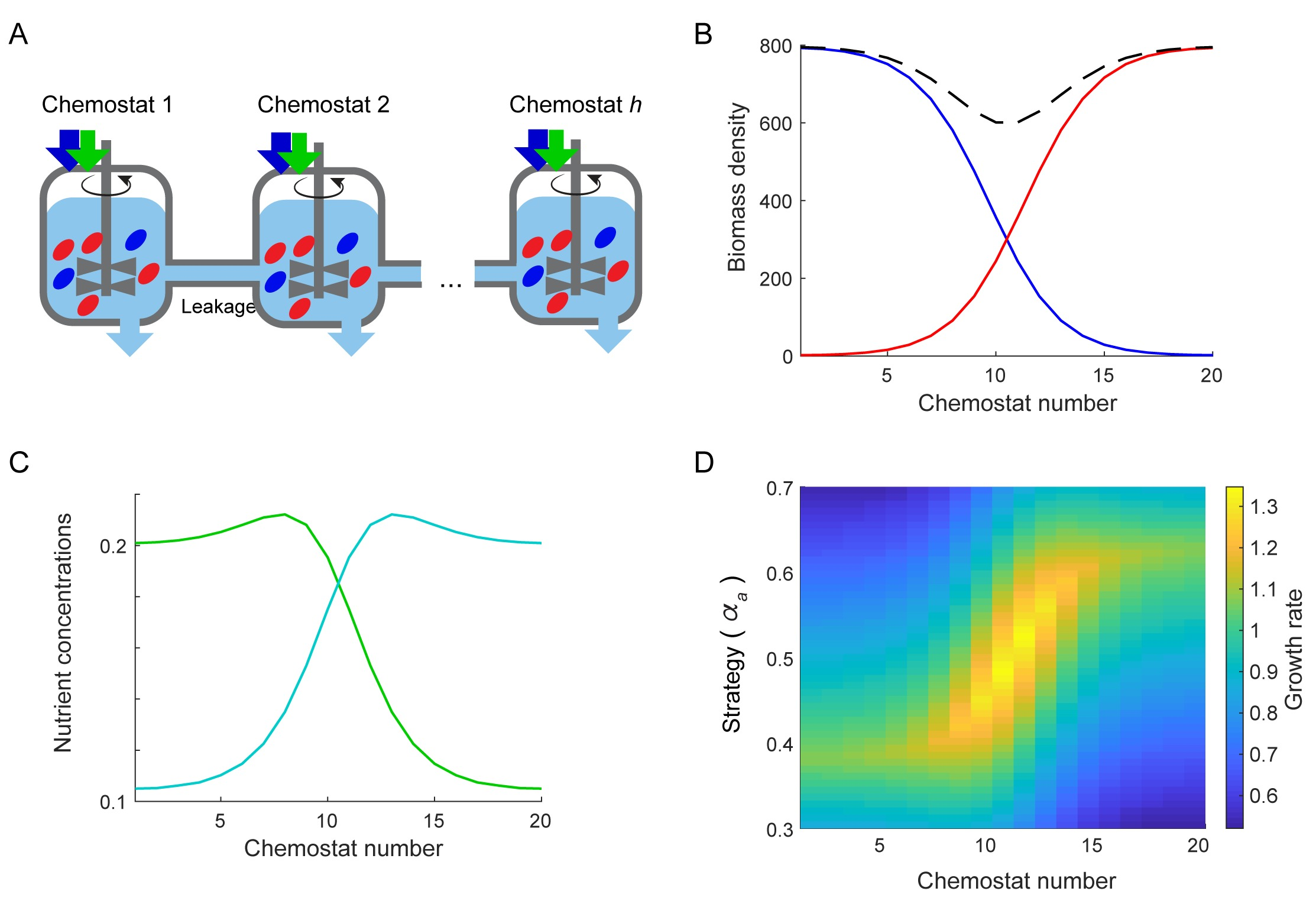

Supplement: S6 Fig — With initial seeding of two species, one at each of the two ends of a chain of chemostats, a steady-state gradient of species biomass density spontaneously emerges accompanied by a gradient of nutrient concentrations, even though the supply conditions and dilution rates are identical for all the chemostats. A. Schematic of ktot linked chemostats exchanging medium and cells via leakage, described by Eqs. S46-S47. The two species in the chemostats (Blue and Red) are the same bistable pair as in Fig 3B and the leakage rate is l = 1. B. The species composition along 20 linked chemostats for the system in (A). Species colors correspond to those in Fig 3B, with species Blue having αa = 0.35 and species Red having αa = 0.65. The dashed black curve shows the sum of the two biomass densities. The initial condition was cell-free chemostats with a small amount of Blue added to Chemostat 1 and small amount of Red added to Chemostat 20. C. Concentrations along the 20 chemostats for nutrient a (green) and nutrient b (cyan) for system in (A). D. The fitness landscape along the chain of chemostats. The x-axis is the 20 linked chemostats, and the y-axis is the metabolic strategy represented by αa. Color indicates the growth rate of species adopting the given strategy in the k-th chemostat. (TIF) [file pcbi.1008156.s007.tif]

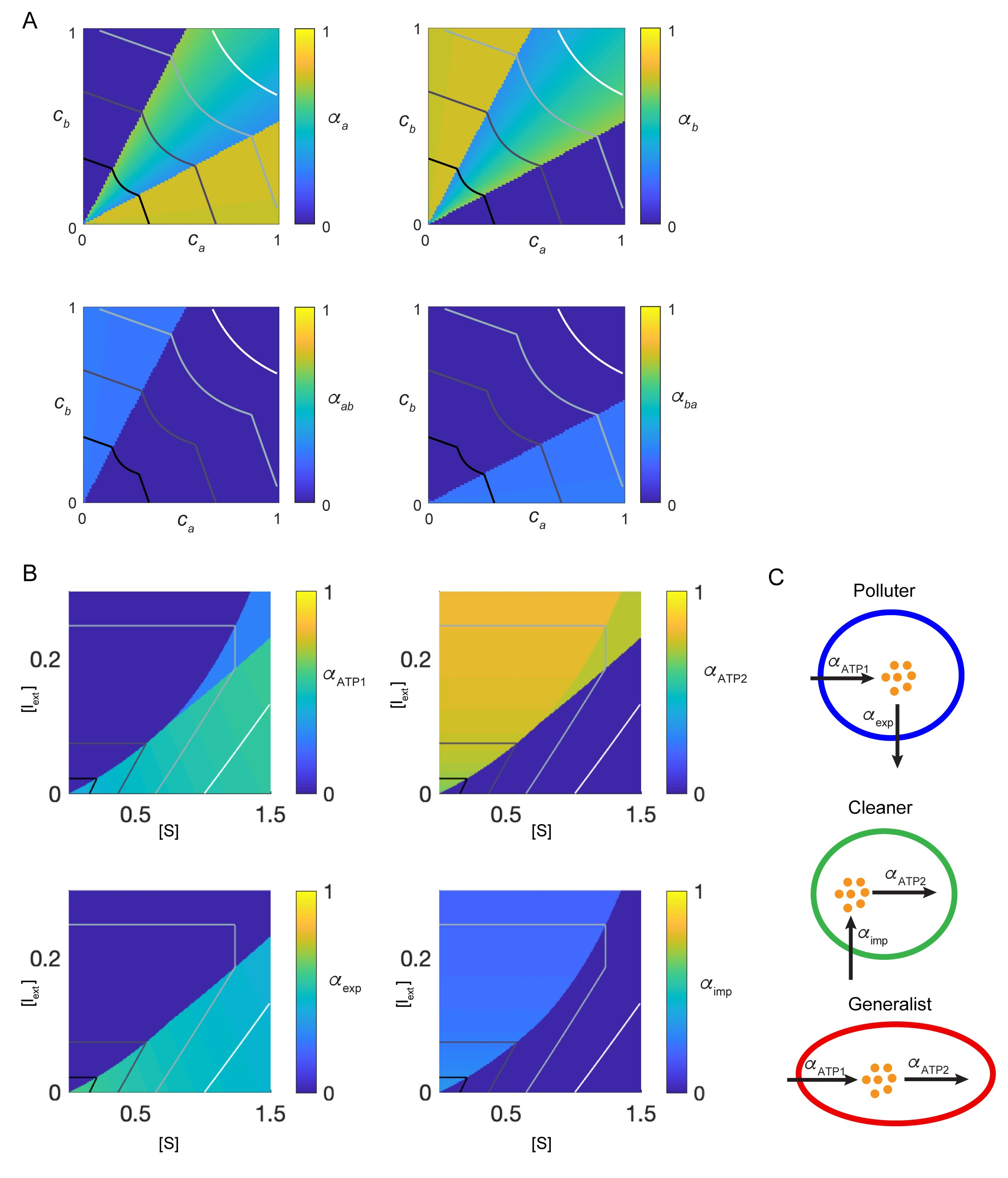

Supplement: S7 Fig — A. For each environment in the chemical space, the maximizing resource allocation strategies that maximize growth rates for the model in Fig 4A. Each strategy is represented by the four elements [αa, αb, αab, αba], and values for each element are shown by a heatmap. Black-to-white curves are the maximal growth contours for d = 0.1, 0.2, 0.3, 0.4. B. For each environment in the chemical space, the maximizing resource allocation strategies that maximize growth rates for the model in Fig 5A. Each strategy is represented by the four elements [αATP1, αATP2, αexp, αimp], and values for each element are shown by a heatmap. Black-to-white curves are the maximal growth contours for d = 0.2, 0.4, 0.6. C. Schematic representations of the three classes of maximizing strategies appearing in (B). (TIF) [file pcbi.1008156.s008.tif]
