# Supplementary material for: Modeling microbial metabolic trade-offs in a chemostat
Source: PLoS Comput Biol. 2020 Aug 28;16(8):e1008156. doi: 10.1371/journal.pcbi.1008156 (PMC7482850; doi:10.1371/journal.pcbi.1008156)
Supplement: Attachment — Submitted filename: Report.pdf [file pcbi.1008156.s009.pdf]

Review report for "Modeling microbial metabolic trade-offs in a chemostat"

In the manuscript, the authors develop a unifying framework that allows to evaluate competing metabolic strategies for different models in chemostat-like systems. Their framework considers the feedback between the influence of the environment on microbial growth and also how microbes shape this environment.

Although the results shown in the manuscript are interesting and could be worthy of publication, there are issues that I think prevent it from being publishable in its present form. These issues, shown below, should therefore be addressed in order to improve the quality of the manuscript and make it more fit for publication.

1. The framework and the graphical tools presented by the authors are interesting and can give useful insights on simple chemostat-like systems, i.e. systems with a limited number of resources. Throughout the manuscript, the authors always show examples where the number of resources is lower or equal to three, since this determines the dimensionality of the "chemical space" and therefore limits graphical representation. It is not clear to me, however, how the framework presented by the authors could be useful when studying more complex systems, i.e. systems with a higher number of resources. I therefore invite the authors to highlight this point in more detail, and possibly add an example where their framework is applied to a system with a larger number of resources (even just in a supplementary file, not necessarily in the main text of the manuscript).
2. By calling $\alpha_{j,\sigma}$ the fraction of internal resources allocated by a species σ to the j-th metabolic function, the authors assume the exact trade-off $\sum_j \alpha_{j,\sigma}=1$. This constraint seems a little bit too strong, since it implies that *all* the internal resources of all species are allocated for metabolism *only*. I think that it would make more sense to use a constraint of the form $\sum_j \alpha_{j,\sigma}=k_\sigma$ with $k_\sigma<1$ some species-dependent constant, so that there is space left for other cellular functions. I invite the authors to investigate how their results change when this assumption is made.
3. Since the authors are describing a chemostat-like system, when writing the equations for the populations (Eq (1)) they use the same dilution rate d for all species. I would expect, however, that there could be some stochasticity in this parameter: even if the microbial species are uniformly distributed in the chemostat, not all of them will be removed at *exactly* the same rate from the system, particularly if the population of some of these species is particularly small. Therefore, I think that it would be interesting to use some dilution rates d_σ drawn from a random distribution, and see how the results of the manuscript change with the variance of this distribution.
4. Explicit results and computations are missing in the "Methods" section. In fact, when the authors show the different metabolic models they use for the simulations they only show the explicit form of the functions I_i and g, and only in one case (substitutable nutrients) they show explicitly the Growth Contour given by the framework. The manuscript would gain remarkably in clarity and completeness if those paragraphs contained, for each metabolic model, the explicit expressions of q_σ and Eqs S4-S6 and S8-S11, i.e. the equations that define the curves (or surfaces) shown in Figures 1-6. In particular, the authors put great emphasis in studying how the population-environment feedback shapes the fitness landscape of a given system, but nowhere in the manuscript is an explicit expression of this landscape for any of the given metabolic models.
5. While the manuscript explains very well why the introduced framework is necessary (i.e., the fact that previous models do not consider the population-environment feedback), it is not clear to me how the conclusions and the results presented by the authors, particularly in terms of invasibility and fitness landscape, relate to the literature and add something new to

what we already know. There are in fact even recent works (like, but not limited to, Dubinkina et al. *eLife* 2019) where people have tried to understand if the introduction of an invader species in an already determined system can result in a successful invasion, and how the invasion could reshape the system. I therefore think the manuscript would gain in completeness if the Discussion contained even a short comparison of the results found by the authors on invasibility and fitness landscape with what we already know.

Finally, I just point out some small typos that I found throughout the manuscript:

- Lines 146 and 149: there should be "indigenous" instead of "endogenous"
- Line 197: there is a missing vector arrow over "q"
- Line 306: there should be "possible" instead of "possibility"
- Line 358: there should be "S4" instead of "S6", and "spontaneous" instead of "spontaneously"
- Line 580: there should be "have been utilizing" or "have utilized" instead of "have been utilized"
- Line 796: there is an extra "{" in $g_{inv}\left(\vec{\alpha}|\vec{c}_{\{\sigma\},ss}\right)$
- Line 1007: there should be "Fig S4A" instead of "Fig S5A"
- Line 1013: there should be "S4" instead of "S5"

References

Dubinkina V., Fridman Y., Pandey P. P. and Maslov S., "Multistability and regime shifts in microbial communities explained by competition for essential nutrients", *eLife* (2019). DOI: 10.7554/eLife.49720
