# Supplementary material for: Modeling microbial metabolic trade-offs in a chemostat
Source: PLoS Comput Biol. 2020 Aug 28;16(8):e1008156. doi: 10.1371/journal.pcbi.1008156 (PMC7482850; doi:10.1371/journal.pcbi.1008156)
Supplement: Attachment — Submitted filename: Response_to_Reviewers.docx [file pcbi.1008156.s010.docx]

**Response to Reviewers**

Reviewer #1:

1. The framework and the graphical tools presented by the authors are interesting and can give useful insights on simple chemostat-like systems, i.e. systems with a limited number of resources. Throughout the manuscript, the authors always show examples where the number of resources is lower or equal to three, since this determines the dimensionality of the “chemical space” and therefore limits graphical representation. It is not clear to me, however, how the framework presented by the authors could be useful when studying more complex systems, i.e. systems with a higher number of resources. I therefore invite the authors to highlight this point in more detail, and possibly add an example where their framework is applied to a system with a larger number of resources (even just in a supplementary file, not necessarily in the main text of the manuscript).

The reviewer’s valuable suggestion to demonstrate how our framework can be applied to systems with chemical dimensions higher than three has led to some interesting updates, which we put in Fig 2E-F, Fig S3, and Fig S5. In brief, the central message of our presented framework, including the geometric “rule of invasion” and the environment-dependent fitness landscape, does not depend on the number of chemical dimensions. Nevertheless, it is informative to apply our framework to systems with a larger number of nutrients to demonstrate how the high-dimensional fitness landscape deforms as “adaptive” species are added. In the substitutable-nutrient and the essential-nutrient models, analytical solutions for the fitness landscapes are readily obtainable (see S1 Appendix). Therefore, we extended these two simple models into10-dimensional chemical space, and studied how the fitness landscape evolves as species and the environment interact with each other: while the essential-nutrient system exhibits a similar “chain of invasion” in both 2-dimensional (Fig 3) and 10-dimensional chemical space (Fig S5), the substitutable-nutrient model with 10 nutrients evolves into a flat fitness landscape (Fig 2E-F, Fig S3), which is not as clear with only 2 substitutable nutrients. We also employed radar plots to qualitatively visualize the high-dimensional growth contours for these two metabolic models (Fig S3D and Fig S5E)

2. By calling αj,σ the fraction of internal resources allocated by a species σ to the j-th metabolic function, the authors assume the exact trade-off ∑ *α j , σ* =1. This constraint seems a little bit too strong, since it implies that *all* the internal resources of all species are allocated for metabolism *only*. I think that it would make more sense to use a constraint of the form ∑*αj,σ*=*kσ* with *kσ*<1 some species-dependent constant, so that there is space left for other cellular functions. I invite the authors to investigate how their results change when this assumption is made.

Indeed, the exact trade-off is a highly simplified assumption. Assuming ∑*α_j,σ_* = *k_σ_* with *k_σ_*<1 is very reasonable for cellular metabolism, and there are two possibilities:

1. *k_σ_* is constant, and species only differ in their strategies in allocating *k_σ_* into different metabolic functions. In this case, *k_σ_* can be identified as the fraction of resources in the metabolic sector, and then rescaled again to 1 without loss of generality.

2. *k_σ_* is variable across different species. That is likely to be more realistic in terms of evaluating ecological outcomes and stability, yet also adds complexity, and makes analytical solutions impossible. Furthermore, considerations of evolutionary stability require an upper limit on internal cellular resources, and so for our current purposes we set *k_σ_* this as an absolute limit shared by all species. We note that a subsequent study following Posfai et al. 2017^[1]^ found that non-exact trade-offs still allow more species than nutrients to coexist if spatial heterogeneity is introduced ^[2, 3]^. In view of these points, we continue use the notation ∑*α_j,σ_* = 1 for simplicity, but discuss the influence of non-exact trade-offs in the Discussion.

3. Since the authors are describing a chemostat-like system, when writing the equations for the populations (Eq (1)) they use the same dilution rate d for all species. I would expect, however, that there could be some stochasticity in this parameter: even if the microbial species are uniformly distributed in the chemostat, not all of them will be removed at *exactly* the same rate from the system, particularly if the population of some of these species is particularly small. Therefore, I think that it would be interesting to use some dilution rates dσ drawn from a random distribution, and see how the results of the manuscript change with the variance of this distribution.

In any ecosystem, random events do occur, not only fluctuations in external conditions such as supply concentrations or dilution rates, but also births and deaths. As the reviewer suggests, stochasticity is most relevant when populations are small. Following this suggestion, we implemented a stochastic simulation in the simple form of an Ito process (with details in S1 Appendix). The rock-paper-scissors model, which leads to periodically very low populations of each species, exhibited the largest deviation between the stochastic and continuous versions, just as suggested by the reviewer. In the stochastic simulation, if we mark a species as “extinct” once its biomass hits zero, the oscillations become unstable. When a species’ number oscillates to its lowest point in a cycle, it may become extinct due to a random fluctuation. Then the system ceases to oscillate and finally ends in a one-species state. This behavior is shown in Fig S4.

4. Explicit results and computations are missing in the “Methods” section. In fact, when the authors show the different metabolic models they use for the simulations they only show the explicit form of the functions Ii and g, and only in one case (substitutable nutrients) they show explicitly the Growth Contour given by the framework. The manuscript would gain remarkably in clarity and completeness if those paragraphs contained, for each metabolic model, the explicit expressions of qσ and Eqs S4-S6 and S8-S11, i.e. the equations that define the curves (or surfaces) shown in Figures 1-6. In particular, the authors put great emphasis in studying how the population-environment feedback shapes the fitness landscape of a given system, but nowhere in the manuscript is an explicit expression of this landscape for any of the given metabolic models.

We thank the reviewer for this good suggestion. We have accordingly revised the supplement to include equations for the steady state, growth contour, fitness landscape, and maximizing strategies in the various metabolic models. (The solution for the metabolic model with multiple energy-generating steps is too long to be put in the text, so it is included in the program package.)

5. While the manuscript explains very well why the introduced framework is necessary (i.e., the fact that previous models do not consider the population-environment feedback), it is not clear to me how the conclusions and the results presented by the authors, particularly in terms of invasibility and fitness landscape, relate to the literature and add something new to what we already know. There are in fact even recent works (like, but not limited to, Dubinkina et al. *eLife* 2019) where people have tried to understand if the introduction of an invader species in an already determined system can result in a successful invasion, and how the invasion could reshape the system. I therefore think the manuscript would gain in completeness if the Discussion contained even a short comparison of the results found by the authors on invasibility and fitness landscape with what we already know.

We have followed the reviewer’s suggestion to better place our results in the context of these recent works. The introduction of an invader species into an existing system has been utilized as a way to assess community stability in several modeling studies^[4, 5]^ particularly in the school of adaptive dynamics ^[6-8]^. In our work, we not only utilized invasion as a tool to evaluate metabolic strategies, but also used the “rule of invasion” together with the deformability of the fitness landscape to assess the outcome of an invasion. Another important difference is that we consider the outcome of invasions among a continuum of strategies, in order to determine the evolutionarily stable strategies, while in most of the other works, invasion is considered between discrete strategies. To highlight these distinctions, we added citations to Dubinkina et al. as well as several other related studies, and discussed their relevance in the Discussion.

Finally, I just point out some small typos that I found throughout the manuscript:

- Lines 146 and 149: there should be “indigenous” instead of “endogenous”
- Line 197: there is a missing vector arrow over “q”
- Line 306: there should be “possible” instead of “possibility”
- Line 358: there should be “S4” instead of “S6”, and “spontaneous” instead of “spontaneously”
- Line 580: there should be “have been utilizing” or “have utilized” instead of “have been utilized”
- Line 796: there is an extra “{“ in *ginv*(⃗*α*∣*c*⃗{*σ*}*,ss*)
- Line 1007: there should be “Fig S4A” instead of “Fig S5A”
- Line 1013: there should be “S4” instead of “S5” **References**

Dubinkina V., Fridman Y., Pandey P. P. and Maslov S., “Multistability and regime shifts in microbial communities explained by competition for essential nutrients”, *eLife* (2019). DOI: 10.7554/eLife.49720

We thank the reviewer for the careful reading – we have corrected all these typos,.

//////////////////////////////////

Reviewer #2:

The authors use an explicit model of resource competition to search for optimal resource allocation strategies in a continuous growth situation, e.g. in a chemostat. They use a general framework—which they have introduced before—that assumes metabolic trade-offs in the form of a linear allocation of limited resources in different metabolic pathways.

The authors start by framing a model and a corresponding graphic visualization to interpret the outcome. They then proceed with a series of examples, with relatively simple and intuitive assumptions, to show different types of complex dynamics that can emerge. The focus throughout the paper is on how the trade-off in allocating resources can lead to ecological dynamics.

The manuscript does a great job of putting its contribution in the context of the existing work. It has a logical flow and is accessible for both general and expert readers, in my opinion (although defining some of the terms in the introduction could help making the paper more readable for non-experts).

I have no major concerns regarding the paper (minor suggestions are listed below).

We thank the reviewer very much for the insightful comments and suggestions. We modified the Introduction and the first section of Results to make the paper more readable for non-experts.

Minor comments:

1. I am not sure if I fully understand what happens in Fig 2. Is the flat landscape a result of a particular choice of parameters for species Blue and Red? Additionally, doesn’t the presence of other species reshape the landscape? Or does the landscape become flat with any combination that has both species with small alpha_a and species with large alpha_a?

The flat landscape is not a result of a particular choice of parameters for species *Blue* and *Red*. As long as *Blue* and *Red* form a mutual-invasion pair and co-create their environment at the intersection of their growth contours, the fitness landscape becomes flat, regardless of the specific strategies of *Blue* and *Red*. The reviewer is correct that any combination of two species with a small $\alpha_{a}$ and a large $\alpha_{a}$ are likely to form a mutual-invasion pair and lead to unlimited coexistence. The specific criterion for mutual invasion is that the two values of $\alpha_{a}$ bracket the fraction of the total nutrient supply that is nutrient 1, i.e. the nutrient supply lies within the “convex hull” of the strategies. This condition extends to higher dimension, and any number of present strategies because of the special analytical property of the substitutable-nutrient model that when the metabolic trade-off is exact ($\sum\alpha_{i}=1$), all growth contours intersect at the same point (S1 Appendix, section *Metabolic model with substitutable nutrients*), which enables a flat fitness landscape. In the updated Fig 2E-F we also showed that for high chemical dimensions, if the species with the highest growth rates sequentially invade, the system progresses towards a flat fitness landscape. We have reorganized and extended the section on the substitutable-nutrient model to make the message clearer.

2. I think it would be helpful to include the list of relevant model parameters and their values in each figure as a point of reference.

This is very helpful suggestion. We have included the relevant list of model parameters and values in each figure.

3. Although conceptually generalizable, the visualization approach works well only with one or two environmental resource (with three resources, as in Fig 3, the visual representation is not nearly as clear). I admit that even with two resources the authors have shown a very rich range of possibilities, but it would be helpful to include your suggestions about how to scale up to more resources.

This point coincides with the first comment of Reviewer #1: despite the fact that the framework in this manuscript, including the geometric “rule of invasion” and the environment-dependent fitness landscape, does not depend on the number of chemical dimensions, it is still worthwhile to show how it works for more nutrient types. Please see our response to the first comment of Reviewer #1, and the updated Fig 2E-F, Fig S3, and Fig S5.

//////////////////////////////////

Reviewer #3: The manuscript presents a very broad exploration of consumer-resource models in constantly diluted chemostats with general functions describing species’ growth rates, nutrient consumption and secretion rates, and their (potentially variable and evolvable) metabolic strategy alpha. The paper adopts the geometric view of Tilman 1982 representing ecosystem dynamics in a multidimensional space of nutrient concentrations inside the chemostat. The scope of the paper is so broad that sometimes it is difficult to separate truly novel results from well-established facts in consumer-resource modeling literature. However, I believe that with a proper narrowing of the scope and streamlining of explanations the paper would be publishable in PLoS Comp Biology. Here are my comments, questions, and recommendations roughly in the order the models were presented in the manuscript:

We thank the reviewer for the important suggestion to clarify which results are novel, and to streamline the manuscript. We have accordingly reorganized the Results and Discussion, and moved the rock-paper-scissors and the control of chemostat experiments sections into S1 Appendix, to make the logical flow smoother and to sharpen the focus of the main text onto evolutionarily stable strategies.

1) Lines 305-306. Invoking the model as an explanation for the paradox of the plankton is rather weak and should be removed. Indeed, a long-term coexistence of species requires near miraculous coincidence: all species have to have exactly the same proteome budget and differ only by their allocation strategies alpha (sum alpha =1). While this is plausible for subpopulations within one genetically identical strain, it is rather unlikely even for closely related strains of the same species (see Lenski experiments and recent studies by Ben Good and Oskar Hallatschek about two types of mutations: one type changing alpha and another type changing the overall growth rate). Given that in the paradox of the plankton a very diverse set of organisms coexists, it is unlikely to be explained by these arguments. In fact, this part of the study is purely pedagogical and does not describe any new results.

The reviewer is right that indefinite coexistence in the substitutable-nutrient model relies on mathematically exact trade-offs. Nevertheless, even approximate trade-offs can promote diversity by partially “leveling the playing field” among different metabolic strategies – allowing for high diversity for low levels of immigration^[1, 3]^ or in the presence of spatial structure ^[2]^. To clarify this point, including the limitations of the exact trade-offs assumption, we have revised the section on the substitutable-nutrient model. This model is presented in the current manuscript primarily to show how the fitness landscape can be flattened by the interplay between species and environment. To this end, we now explicitly demonstrate how the invasion of species with maximal growth rates in the current environment leads to self-organization of the community to create a flat landscape for all species.

2) Lines 336-337. The model described in this section is potentially new and interesting. However, I did not understand from the main text how exactly a mismatch between allocation towards nutrient import and conversion can lead to rock-paper-scissors oscillations. If it is described in SI, it should be moved to the main text. How is this model different from Huisman et al 1999, especially from a more detailed paper by the same authors: Jef Huisman and Franz J Weissing, ECOLOGY 2001 “Biological conditions for oscillations and chaos generated by multispecies competition”?

The mismatch between allocation towards nutrient import and conversion works as following: species 1 (*Red*, $\alpha_{c1}$ > $\alpha_{a1}$ > $\alpha_{b1}$, $\alpha_{b2}\sim\alpha_{a2}>\alpha_{c2}$) is good at importing nutrient *c* but favors an environment with a high concentration of *a* (see Fig S4B). Therefore, in the environment created by species 1, nutrient *c* is low while *b* is high. This environment is inviting for species 2 (*Green*) which is good at importing *a* but prefers *b*, but not for species 3 (*Blue*) which is good at importing *b* but prefers *c*. Such a mismatch between “imported nutrients” and “preferred nutrients” allows for the rock-paper-scissors loop of invasion, and promotes oscillations. As this model addresses essentially the same mechanism presented in the work of Huisman and Franz, we have provided the above clarifying explanation and moved the “rock-paper-scissors” section into S1 Appendix. This has the additional advantage of focusing the main text more narrowly on evolutionarily stable strategies.

3) The part of the manuscript describing multistability needs to be put in context of a recent 2019 paper from Maslov lab dedicated exclusively to this topic. What are authors’ new insights into the topic of multistability? Also, it has not been spelled very clearly in this manuscript that in MacArthur type models multistability is possible only when different species have different yields on the same nutrient. This clear and simple message has been lost in the general approach adopted in this manuscript.

We thank the reviewer for the good suggestion to clarify which of our results regarding multistability are novel, and what are the underlying requirements. As a key assumption of the essential-nutrient metabolic model, growth rate is constrained by the least abundant resource, the stoichiometry ratio is therefore different for each strategy, satisfying the accepted criterion that strategies must have different yields on the same nutrients to enable multistability. In our work, we aim to show the necessary and sufficient criteria for multistability from the perspective of the dynamic fitness landscape: species placing themselves near the top of the fitness landscapes they generate.

In most of Maslov and colleagues’ studies of multistability, the strategies are discrete. In our work, we further demonstrate that when species are allowed to evolve in a continuous strategy space, a large number of strategies that cannot invade each other emerge as candidates for multistability. This metabolic model is useful to highlight the difference between ecological stability and evolutionary stability: small adaptive changes in $\alpha_{a}$ lead to a chain of invasion, producing a large number of pairs of species capable of multistability, while ultimately leading to an evolutionarily stable “non-invasible” strategy. In the revised version, we discuss the relation of our work with the multistability studies from Maslov and colleagues in the Discussion.

4) Evolutionary stable strategies resisting invasion by other species with different metabolic strategies is a truly novel ingredient of this manuscript (see though a recent preprint from Martian and Suweis labs with similar discussion). However, at this point in the manuscript I was so exhausted plowing through previous results that I did not understand the main point authors are trying to make here. I would like authors to sharpen their message in this section. They should also put their results in context of Tikhonov and Monasson 2018 J Stat Phys paper on evolution of parameters in consumer-resource models.

Following these suggestions, we have accordingly reorganized the Results and Discussion sections to sharpen the focus on evolutionary stable strategies, and also compare our message with other relevant studies. In the work from the Maritan and Suweis's labs “Dynamic metabolic adaptation can promote species coexistence in competitive microbial communities” recently published in PLOS Comp Biol ^[9]^, adaptive metabolic strategies in the framework of a consumer-resource model were introduced, where species are allowed to adjust their metabolic strategies over time to maximize their relative fitness. The metabolic model they used is a linear summation model, similar to that of Posfai et al. ^[1]^ and they also used the convex hull formulation. The main focus of that study is the growth of a single microbial species on multiple resources (diauxic shifts) and how changing supplies influence the community. An interesting finding in their “Species coexistence” section is that “…we show the initial and final states of a temporal integration of the model: even though [the supply] was initially outside the convex hull…, the metabolic strategies changed to bring [the supply] within the convex hull and thus allowed coexistence.” In our Discussion, we now cite this result as “In a recent publication ^[9]^, species are allowed to adjust their metabolic strategies over time to maximize their relative fitness. These adaptive strategies yield similar results to our evolutionary adaptation via invasion: even when the supply condition initially does not favor coexistence, the adaptation of metabolic strategies self-organizes the population into a state of coexistence.”

These results complement our observation in the 10-dimensional case, that invasions by the fastest growing strategies drives the system towards a flat fitness landscape.

5) The discussion of creating metabolic niches by secreting metabolic byproducts is not new. What is missing is the discussion of authors insights in context of Marsland et al “Available energy fluxes drive a transition in the diversity, stability, and functional structure of microbial communities” and Goyal et al PRL 2018.
I would be happy to review a more streamlined version of this manuscript again.

As the reviewer points out, there are many modeling works focusing on coexistence based on species creating metabolic niches by secreting metabolic byproducts. Our particular question concerns the evolutionary stability of such coexistence: can the ecological coexistence survive mutation and selection? As we write “Why doesn’t the producer in a cross-feeding pair adjust its strategy to retain all useful metabolites? And can evolutionarily stable coexistence occur due to secreted metabolites even without mutually beneficial cross-feeding?” Among the several types of evolutionarily stable coexistence we investigated, the most unexpected is between a “polluter” and a “generalist”, instead of between a “polluter” and a “cleaner”, demonstrating that metabolite secretion can lead to diversity even in the absence of cross-feeding. This observation complements and extends the previous consensus that coexistence can be enabled by unilateral or mutually beneficial cross-feeding ^[6, 10-12]^. We have clarified these points and added a discussion related to the work of Goyal et al. ^[13]^ and et al. ^[10]^ in the Discussion.

1.	Posfai A, Taillefumier T, Wingreen NS. Metabolic Trade-Offs Promote Diversity in a Model Ecosystem. Physical Review Letters 2017, **118**(2).

2.	Weiner BG, Posfai A, Wingreen NS. Spatial ecology of territorial populations. Proceedings of the National Academy of Sciences 2019, **116**(36)**:** 17874-17879.

3.	Erez A, Lopez JG, Weiner B, Meir Y, Wingreen NS. Nutrient levels and trade-offs control diversity in a model seasonal ecosystem. arXiv preprint arXiv:190209039 2019.

4.	Tikhonov M, Monasson R. Innovation rather than improvement: A solvable high-dimensional model highlights the limitations of scalar fitness. Journal of Statistical Physics 2018, **172**(1)**:** 74-104.

5.	Pacciani-Mori L, Suweis S, Maritan A, Giometto A. Adaptive metabolic strategies in consumer-resource models. bioRxiv 2019**:** 385724.

6.	Doebeli M. A model for the evolutionary dynamics of cross-feeding polymorphisms in microorganisms. Popul Ecol 2002, **44**(2)**:** 59-70.

7.	Ispolatov I, Madhok V, Doebeli M. Individual-based models for adaptive diversification in high-dimensional phenotype spaces. Journal of Theoretical Biology 2016, **390:** 97-105.

8.	Rueffler C, Van Dooren TJM, Metz JAJ. Adaptive walks on changing landscapes: Levins' approach extended. Theoretical Population Biology 2004, **65**(2)**:** 165-178.

9.	Pacciani-Mori L, Giometto A, Suweis S, Maritan A. Dynamic metabolic adaptation can promote species coexistence in competitive communities. PLoS computational biology 2020, **16**(5)**:** e1007896.

10.	Marsland III R, Cui W, Goldford J, Sanchez A, Korolev K, Mehta P. Available energy fluxes drive a transition in the diversity, stability, and functional structure of microbial communities. PLoS computational biology 2019, **15**(2)**:** e1006793.

11.	Pfeiffer T, Bonhoeffer S. Evolution of cross-feeding in microbial populations. The American naturalist 2004, **163**(6)**:** E126-E135.

12.	Goldford JE, Lu N, Bajić D, Estrela S, Tikhonov M, Sanchez-Gorostiaga A, Segrè D, Mehta P, Sanchez A. Emergent simplicity in microbial community assembly. Science 2018, **361**(6401)**:** 469-474.

13.	Goyal A, Maslov S. Diversity, stability, and reproducibility in stochastically assembled microbial ecosystems. Physical Review Letters 2018, **120**(15)**:** 158102.
